# Supplementary material for: North Andean origin and diversification of the largest ithomiine butterfly genus
Source: Sci Rep. 2017 Apr 7;7:45966. doi: 10.1038/srep45966 (PMC5384087; doi:10.1038/srep45966)
Supplement: Supplementary Information [file srep45966-s1.pdf]

North Andean origin and diversification of the largest ithomiine butterfly genus

Donna Lisa De-Silva[1], Luísa L. Mota[2], Nicolas Chazot[1,3], Ricardo Mallarino[4], Karina L. Silva-Brandão[5], Luz Miryam Gómez Piñerez[6,7], André V.L. Freitas[2], Gerardo Lamas[8], Mathieu Joron[9], James Mallet[4], Carlos E. Giraldo[6], Sandra Uribe[6], Tiina Särkinen[10], Sandra Knapp[11], Chris D. Jiggins[12], Keith R. Willmott[13,] Marianne Elias*[1]

1. Institut de Systématique, Évolution, Biodiversité, ISYEB - UMR 7205 – CNRS MNHN UPMC EPHE, Muséum National d'Histoire Naturelle, Sorbonne Universités, 57 rue Cuvier CP50 F-75005, Paris, France.
2. Departamento de Zoologia and Museu de Zoologia, Instituto de Biologia, Universidade Estadual de Campinas, Campinas, São Paulo, Brazil.
3. Department of Biology, Lund University, Lund, Sweden
4. Department of Organismic and Evolutionary Biology, Harvard University, Cambridge, MA 02138, USA.
5. Centro de Biologia Molecular e Engenharia Genética, Universidade Estadual de Campinas, Campinas, São Paulo, Brazil
6. Universidad Nacional de Colombia, sede Medellín, Medellín, Colombia.
7. Grupo de investigation Ciencias Forenses y Salud, Tecnológico de Antioquia,
8. Museo de Historia Natural, Universidad Nacional Mayor de San Marcos, Lima, Peru.
9. Centre d'Ecologie Fonctionnelle et Evolutive, CEFE, UMR 5175 CNRS - EPHE - Université de Montpellier - Université Paul Valéry Montpellier, 34293 Montpellier 5, France.
10. Royal Botanic Garden Edinburgh, 20A Inverleith Row, Edinburgh EH3 5LR, U.K.
11. Department of Life Sciences, Natural History Museum, Cromwell Road, London SW7 5BD, U.K.
12. Department of Zoology, University of Cambridge, Cambridge, U.K.
13. McGuire Center for Lepidoptera and Biodiversity, Florida Museum of Natural History, University of Florida, Gainesville, Florida 32611, USA.

Supplementary method S1: (1) list and description of morphological characters, (2) recalibration of the Solanaceae phylogeny.

1- list and description of morphological characters

Male Genitalia

Aedeagus

1. Aedeagus with triangular dorsal spine near posterior tip: (0) absent; (1) present. State 1 is a synapomorphy for P. zerlina and relatives.

2. Aedeagus with sclerotized 'ribbon' extending dorsally from anterior tip along anterior edge of ductus ejaculatorius: (0) absent; (1) present. State 1 occurs only in *P. donella* and *P. hara*.

3. Aedeagus with laterally flaring flanges on both sides near posterior tip: (0) absent; (1) present. State 1 occurs only in *P. olimba*.

4. Aedeagus width: (0) greater in anterior half; (1) even throughout.

5. Aedeagus with small, posteriorly directed dorsal 'teeth' near posterior tip: (0) absent; (1) present. State 1 occurs only in *P. sao* and *P. obscuratus*.

6. Aedeagus with lateral projections at anterior tip: (0) short (less than width of aedeagus anterior tip); (1) long (similar to or greater than width of aedeagus anterior tip).

7. Aedeagus in lateral view with anterior tip: (0) straight or smoothly curving with respect to adjacent aedeagus; (1) bent sharply upwards.

8. Aedeagus with ductus ejaculatorium: (0) close to anterior tip (anterior portion of aedeagus beyond ductus ejaculatorium less than or similar to aedeagus width); (1) displaced posteriorly from anterior tip (anterior portion of aedeagus beyond ductus ejaculatorium much longer than aedeagus width).

9. Aedeagus with posterior portion: (0) similar in length to posterior portion (posterior to zone); (1) much shorter than anterior portion (approximately half length).

10. Posterior portion of aedeagus: (0) straight or slightly curved downwards (continuously variable); (1) curved upwards; (2) sharply bent downwards near posterior tip.

11. Anterior portion of aedeagus: (0) straight or slightly curved downwards (continuously variable); (1) curving smoothly upwards (1).

12. Anterior portion of aedeagus angled with respect to posterior portion: (0) parallel; (1) downwards; (2) upwards.

Gnathos

13. Gnathos: (0) present; (1) absent. State 1 occurs only in *P. sao*, *P. obscuratus*, *P. tucuna* and *P. latilla*.

14. Gnathos 'arms': (0) of even width; (1) restricted laterally in middle.

15. Gnathos ventral portion in ventral view: (0) with narrow, posteriorly directed projection; (1) with broad, triangular, anteriorly directed projection; (2) of similar width to gnathos 'arms'; (3) with both anteriorly and posteriorly directed, broad projections; (4) with single, broad posteriorly directed projection; (5) with two posteriorly directed projections.

Uncus

16. Uncus length: (0) short (less than half of to similar in length to tegumen); (1) long (same size or larger than tegumen).

Tegumen

17. Angle between tegumen and vinculum in lateral view: (0) acute (less than 90º); (1) obtuse (90º or more).

Saccus

18. Saccus length: (0) greater than that of vinculum (<2x length vinculum); (1) less than that of vinculum; (2) much greater than that of vinculum (>2x length vinculum).

Valva

19. Sclerotized 'rib' along anterio-dorsal edge of valva: (0) present; (1) absent.

20. Valva inner ventral edge in ventral view: (0) straight or forming a smooth convex curve; (1) with inner projections near middle; (2) parallel in basal half then curved inwards near posterior tip; (3) smoothly concave throughout.

21. Tip of valva ventral inner projections (char. 20:1): (0) smooth point; (1) indented in middle.

22. Valvae dorsal inner projections: (0) symmetrical; (1) asymmetrical.

23. Right-hand valva dorsal inner projection with tip: (0) rounded; (1) sharp.

24. Valvae dorsal inner projections in ventral view: (0) not or barely overlapping with gnathos; (1) substantially overlapping with gnathos.

Juxta

25. Juxta in lateral view: (0) straight or lightly curving; (1) distinctly curving with concave side directed anteriorly.

26. Valva shape in lateral view: (0) approximately trapezoidal with posterior tip a rounded point; (1) with a ventrally indented edge and squared posterior tip; (2) with a ventrally indented edge and with posterior tip a rounded point; (3) with a smoothly curving ventral edge and posterior tip a sharp, narrow point.

Male wing venation

Hindwing

27. Origin of vein M3: (0) more proximal than origin of vein Rs+M1; (1) as distal or more distal than origin of vein Rs+M1.

28. Veins Rs+M1: (0) not fused; (1) totally fused; (2) fused partly but bifurcating distally.

29. If Vein M1 and Rs are partially fused (char. 28:2): (0) vein M1 arises proximally (before midpoint of veins); (1) vein M1 arises distally (beyond midpoint of veins).

30. Hair-like androconial scales on dorsal edge along costa: (0) only in basal half of wing; (1) continually distributed throughout costa; (2) throughout costa but partially or completely broken into two patches.

31. Wing membrane at proximal area of costal androconial scales: (0) deeply grooved; (1) almost flat.

32. Basal blade-like androconial scales underlying hair-like scales at dorsal costa orientated with respect to wing membrane: (0) vertically; (1) horizontally.

33. Basal blade-like androconial scales underlying hair-like scales at dorsal costa color: (0) varying from black or brown to gray; (1) white.

34. Basal blade-like androconial scales underlying hair-like scales at dorsal costa color compared to distal blade-like androconial scales: (0) different color; (1) same color.

35. Distal blade-like androconial scales underlying hair-like scales at dorsal costa shape: (0) rounded at tip; (1) pointed at tip.

Female Genitalia

36. Ostium bursae size: (0) medium (approximately 25% width 7th sternite); (1) small (<25% width 7th sternite); (2) large (approximately 50% width 7th sternite); (3) very large (>50% width 7th sternite).

37. Ostium bursae shape and sclerotization: (0) a short, even, sclerotized tube similar in length to width, and similar in width to ductus bursae; (1) ductus bursae apparently connects directly to 8th sternite plates, no sclerotized or modified ostium bursae; (2) a short sclerotized tube curved to left side; (3) a weakly sclerotized, broad, tapering cone-shaped tube, sclerotized more strongly dorsally; (4) a weakly sclerotized tube with two dorsal sclerotized plates; (5) a very large, weakly sclerotized, uneven, hemispherical tube; (6) a short, tapering sclerotized tube; (7) a weakly sclerotized, ribbed, tapering tube; (8) a short, tapering, weakly sclerotized tube; (9) a long, broad, sclerotized, even tube; (A) a short, broad, sclerotized (more weakly dorsally), even tube; (B) a sclerotized, broad, tapering cone-shaped tube.

38. Ductus bursae width: (0) more than one quarter of the 7th sternite's width; (1) less than one quarter of the 7th sternite's width.

39. Connection between corpus bursae and appendix bursae: (0) narrow; (1) broad.

40. 8th sternite formed by: (0) a single plate; (1) two plates.

41. Ostium bursae position: (0) opposite middle of 7th sternite; (1) dislocated to left of middle of 7th sternite.

42. 8th sternite lip sclerotization: (0) sclerotized; (1) not sclerotized.

43. Posterior edge of 7th sternite: (0) smoothly curving or straight; (1) indented around ostium bursae. This character was only coded for species with a small or medium-sized ostium bursae.

44. 8th sternite plates: (0) not extending anteriorly around ostium bursae; (1) extending anteriorly to encircle ostium bursae.

Adult Abdomen

45. Color of the adult ventral abdomen: (0) yellow; (1) white or mixture of white and black.

Immature Stages

Larvae

46. Cephalic capsule with black pigmentation: (0) absent; (1) present.

47. If there is black pigmentation in the cephalic capsule, it is arranged as: (0) two major lateral spots; (1) two horizontal lines; (2) many spots; (3) a continuous spot covering all of head.

48. White pigmentation forming an inverted “v” in the frontal cephalic capsule: (0) absent; (1) present.

49. Beige pigmentation in the cephalic capsule: (0) absent; (1) present.

50. Black pigmentation in the body: (0) present; (1) absent.

51. If there is black pigmentation in the body, it forms: (0) small dorsal spots; (1) dorsal stripes (complete or not complete).

52. If there are dorsal black stripes on the body (char. 51:1), they are: (0) narrow, occupying one quarter of a segment; (1) broad, occupying one half of a segment;

53. If there are narrow black dorsal stripes on the body (char. 52:0), they: (0) cross the entire segment; (1) are broken in the middle of the body.

54. Dorsal white pigmentation apart from longitudinal central stripe: (0) absent; (1) present.

55. If there is dorsal white pigmentation, apart from central circular spots and sub-dorsal longitudinal stripes, it appears as: (0) narrow transverse stripes, each occupying less than one quarter of a segment; (1) broad transverse stripes, each occupying one half of the segment.

56. If there are narrow transverse white stripes (char. 55:0), they are: (0) interrupted in the middle of the dorsal area of the segment; (1) restricted to the middle of the dorsal area of the segment.

57. Longitudinal pale dorsal stripe: (0) absent; (1) present.

58. Longitudinal sub-dorsal stripes: (0) absent; (1) present.

59. Longitudinal lateral stripes: (0) absent; (1) present.

60. If there are lateral longitudinal stripes (char. 59:1), they are: (0) yellow/orange; (1) pale green; (2) white.

61.Sub-ventral longitudinal pale stripes: (0) absent; (1) present.

62. Prothorax shape: (0) not enlarged; (1) slightly enlarged.

63. If the prothorax is enlarged (char. 62:1), its color is: (0) the same as in other segments; (1) different, forming a contrasting collar.

64. 8th abdominal segment shape relative to adjacent segments: (0) not enlarged; (1) enlarged.

65. If 8th abdominal segment is enlarged (char. 64:1), its color is: (0) modified only in lateral areas; (1) modified, forming a complete belt only in last sub-segment; (2) modified, forming a complete belt in all sub-segments.

66. Clear pigmentation forming a circle between two segments in the dorsal region: (0) absent; (1) present.

67. If such circles are present as in char. 66:1, their color is: (0) white; (1) orange.

68. Clear pigmentation forming an '8'-shape between 2 segments in the dorsal area: (0) absent; (1) present.

69. Black pigmentation posterior to 8th abdominal segment: (0) absent; (1) present.

70. If there is black pigmentation posterior to 8th abdominal segment, it appears as: (0) a single line; (1) covering whole anal shield; (2) two spots.

71. Last instar ventral color: (0) green; (1) orange. (move to correct place in matrix

Pupa

72. Abdominal silver markings in dorsal view: (0) on all segments; (1) absent on segments A1 to A3.

73. When abdominal silver markings are present on all segments (char. 71:1), markings in dorsal view are: (0) uninterrupted; (1) interrupted in middle; (2) with two interruptions, forming a middle silver line.

74. Lateral part of metathorax: (0) forming a protuberance; (1) even throughout.

75. Mesothoracic protuberance in dorsal view: (0) forming an angle of 90º; (1) forming an angle of less than 90º.

76. Pre-ocular area with: (0) round protuberance; (1) triangular protuberance; (2) no protuberance.

77. Mesothoracic protuberance color: (0) silver; (1) partially silver; (2) not silver.

78. Pre-ocular protuberance color: (0) silver; (1) not silver.

79. Silver marking in the wing cover: (0) highlighting venation; (1) covering wing area or at least wing borders; (2) restricted to apical area; (3) restricted to distal margin and costa.

80. Brown stripes in wing cover: (0) absent; (1) present.

81. Color of cremaster stalk: (0) colorless; (1) red; (2) black.

82. Abdominal segment 1 in comparison to segment 2: (0) constricted to half or less width; (1) of similar width.

83. Protuberances at base of the cremaster stalk in dorsal view: (0) conspicuous; (1) absent or vestigial.

84. Pupal angle between cremaster, ventral abdominal segment 4 and head: (0) 90º or more; (1) less than 90º .

85. Mesothorax in lateral view: (0) rounded; (1) pointed.

86. Pupal principal ground color: (0) green; (1) brown.

Adult Sphragis

87. Sphragis in mated females: (0) absent; (1) present.

2- **Beast dated Solanaceae phylogeny**. The new dated phylogeny was generated in BEAST 1.8.2 (Drummond et al., 2012). We used the sequence matrix of (Särkinen et al., 2013), which comprises 7 genes, totalling 8738 nucleotides. We used (Särkinen et al., 2013)'s original partition and substitution models (7 regions with GTR+G each). An uncorrelated relaxed lognormal clock and a Birth-Death tree prior were used. A uniform prior bounded between 86.9 and 47.8 and with a starting value of 66.6 (values extracted from (Magallón et al., 2015) was imposed on the age of the divergence between the Solanaceae and their sister clade Convolvulaceae (represented by the genus *Ipomoea*). (Särkinen et al., 2013)'s phylogeny was used as a starting tree, after re-scaling with PATHd8 (Britton et al., 2007) to match the age interval of the prior. Two runs of 38 and 31-million generations were performed on the CIPRES server (Miller et al., 2010) (the runs were set to last 50-million generation, but they were interrupted after one week, the running time limit on CIPRES), and the result files were combined using logCombiner (Drummond et al., 2012) with a 10% burnin, after checking for convergence. The maximum credibility clade with median branch length was extracted with TreeAnnotator (Drummond et al., 2012).

Supplementary references

Britton, T., Anderson, C.L., Jacquet, D., Lundqvist, S., Bremer, K., 2007. Estimating divergence times in large phylogenetic trees. Syst. Biol. 56, 741-752.

Drummond, A.J., Suchard, M.A., Xie, D., Rambaut, A., 2012. Bayesian Phylogenetics with BEAUti and the BEAST 1.7. Molecular Biology and Evolution 29, 1969-1973.

Magallón, S., Gomez-Acevedo, S., Sanchez-Reyes, L.L., Hernandez-Hernandez, T., 2015. A metacalibrated time-tree documents the early rise of flowering plant phylogenetic diversity. New Phytologist 207, 437-453.

Miller, M.A., Pfeiffer, W., Schwartz, T., 2010. Creating the CIPRES Science Gateway for Inference of Large Phylogenetic Trees. . SC10 Workshop on Gateway Computing Environments (GCE10).

Särkinen, T., Bohs, L., Olmstead, R.G., Knapp, S., 2013. A phylogenetic framework for evolutionary study of the nightshades (Solanaceae): a dated 1000-tip tree. BMC Evol. Biol. 13.

Supplementary table S1. List of all specimens and outgroups, with GenBank accession numbers for each gene

	species and subspecies name	former species name	code	mtDNA	EF1a	tektin	Locality
***Pteronymia* specimens**							
	Pteronymia aletta lilla		20059	KY750031			Ecuador:
	Pteronymia aletta lilla		E-26-1	DQ157537	DQ177982		Ecuador: Esmeraldas, Esmeraldas
	Pteronymia aletta sspnov		PT032	KY750030	KY750124	KY749868	Colombia: Antioquia Ciudad Bolivar - "Terranova"
	Pteronymia aletta sspnov 1		8879	KY750032	KY750125	KY749869	Panama: Cana, around runway area
	Pteronymia alida sspnov 5		ME10-321	KY750035		KY749873	Ecuador: Napo, Yanayacu
	Pteronymia alida sspnov 6		21025	KY750033	KY750126	KY749870	Ecuador: Zamora-Chinchipe, San Francisco
	Pteronymia alida sspnov 6		21093	KY750034	KY750127	KY749871	Ecuador: Zamora-Chinchipe, San Francisco
	Pteronymia alida sspnov 6		21185	JX573812	JX573739	KY749872	Ecuador: Zamora-Chinchipe, San Francisco
	Pteronymia alida sspnov 6		MJ07-620	KY750000		KY749874	Peru: AM, Molinopampa
	Pteronymia alina machay	*zerlina*	20056	KY750108	KY750200	KY749980	Ecuador:
	Pteronymia alina machay	*zerlina*	21224	KY750109	KY750201	KY749981	Ecuador: Zamora-Chinchipe, San Francisco
	Pteronymia alina machay	*zerlina*	21225	JX573825	JX573752	KY749982	Ecuador: Zamora-Chinchipe, San Francisco
	Pteronymia alina sspnov 1	*zerlina*	21598	KY750110		KY749983	Peru: CU, San Pedro
	Pteronymia alissa alissa		LEP-06912	KY750118			Ecuador: West Carchi 'Cascada Pileta'
	Pteronymia alissa alissa		LEP-08684	KY750029			Ecuador: West Carchi 'Río Chorro Blanco'
	Pteronymia andreas andreas	*alissa*	20043	KY750036	KY750128	KY749875	Ecuador:
	Pteronymia andreas andreas	*alissa*	20046	KY750037	KY750129	KY749876	Ecuador: Morona-Santiago, Yakunk-Cutucu trail, river camp
	Pteronymia andreas andreas	*alissa*	21323	KY750038	KY750130	KY749877	Ecuador: Zamora-Chinchipe, San Francisco
	Pteronymia andreas andreas	*alissa*	02-765	KY750040		KY749879	Peru: San Martín, Chumia, Km14 Shapaja - Chazuta
	Pteronymia andreas andreas	*alissa*	21607	KY750039	KY750131	KY749878	Peru: CU, San Pedro
	Pteronymia andreas dorothyae	*alissa*	LEP-01287	KY750119			Venezuela:
	Pteronymia artena afrania		20039	KY750041	KY750132	KY749880	Ecuador: Morona-Santiago, Loma Kilamo , Macas
	Pteronymia artena afrania		20040	KY750042	KY750133	KY749881	Ecuador: Morona-Santiago, Yakunk-Cutucu trail, lower ridge
	Pteronymia artena afrania		21565	KY750043		KY749882	Peru: Cuzco, San Pedro
	Pteronymia artena afrania		ME10-370	KY750044		KY749883	Ecuador: Napo, Cocodrilo
	Pteronymia artena sspnov 1		8371	KY750045		KY749884	Panama: Fortuna, Quebrada Hornito Trail
	Pteronymia asopo asopo	*oneida*	21169	KY750046	KY750134	KY749885	Venezuela: Andean Region, Rancho Grande
	Pteronymia carlia		BAKU18	KY750047	KY750135	KY749886	Brazil: Minas Gerais, Alto Caparao, Parque Nacional de Caparao, Base alto Caparao
	Pteronymia carlia		MJ07-673	KY750117			Brazil: Paraná, Morretes (Alto da Serra)
	Pteronymia cotytto		20999	KY750052	KY750138	KY749891	Central America:
	Pteronymia cotytto cotytto		MJ07-626	KY750028			Nicaragua: Matagalpa Km 146 Mataglapa-Jinotega
	Pteronymia dispar dispar	*picta*	LEP-11330	KY750027			Colombia: Antioquia
	Pteronymia dispar dispar	*picta*	PT010	KY750123			Colombia: Antioquia Ciudad Bolivar - "Las Acacias- F1"
	Pteronymia dispar dispar	*picta*	PT033	KY750054	KY750139	KY749894	Colombia: Antioquia Fredonia - "San Cayetano" F1
	Pteronymia donella donata		MJ07-629	KY750024			Panama: Darien, Cana Cerro Pirre
	Pteronymia donella donata		MJ07-630	KY750026			Panama: Darien, Cana Cerro Pirre
	Pteronymia donella donella		MJ07-632	KY750023			Colombia: Cundinamarca, 23.6km W of Villeta
	Pteronymia donella donella		PT001	KY750055	KY750140	KY749895	Colombia: Antioquia Amalfi "Porce - El Caimán"
	Pteronymia donella donella		PT025	KY750005	KY750141	KY749896	Colombia: Antioquia Amalfi "Porce - El Encanto"
	Pteronymia donella donella		PT026	KY750025	KY750142	KY749897	Colombia: Antioquia Amalfi "Porce - El Caimán"
	Pteronymia euritea		BAKU1	KY750219		KY749898	Brazil: Minas Gerais, Alto Caparao, Parque Nacional de Caparao, Base alto Caparao
	Pteronymia euritea		MJ07-633		KY750204		Brazil: Espirito Santo, Itaguaçu
	Pteronymia forsteri		02-1429	KF268429	KF268428	KY749901	Peru: San Martín, Chumia, Km14 Shapaja - Chazuta
	Pteronymia forsteri		02-216	KY750058	KY750145	KY749902	Peru: San Martín, Chumia, Km14 Shapaja - Chazuta
	Pteronymia forsteri		05-264	KY750056	KY750143	KY749899	Peru: Loreto, PNCAZ, Pongo del Río Pauya
	Pteronymia forsteri		05-771	KY750057	KY750144	KY749900	Peru: Loreto, PNCAZ, Quebrada Yanayacu (Camp 1), nr Robashca, Puesto de Control 11
	Pteronymia forsteri		BAKU10	KY750059		KY749903	Brazil: Acre, Marechal Thaumaturgo, Res. Extr. Alto Rio Jurua, Colocaçao Pedra Pintada
	Pteronymia fulvimargo		MJ07-640	KY750021			Panama: Chiriqui, Boquete, San Ramón
	Pteronymia gertschi		20072	KY750060	KY750146	KY749904	Ecuador:
	Pteronymia granica		165	KY750061	KY750147	KY749905	Ecuador: Loja, Vilcabamba Ecolodge, Eastern Trail from Ruinas Lodge to Rumi Wilco
	Pteronymia granica		LEP-01505	KY750062		KY749906	Ecuador: Loja, Santuario San Vicente, Quebrada El Sauce, 2km W San Pedro de la Bendita
	Pteronymia hara hara		20035	KY750063	KY750148	KY749907	Ecuador: Zamora-Chinchipe, San Francisco, casa de Arcoiris, km 23 Loja-Zamora rd.
	Pteronymia hara hara		20075	KY750064	KY750149	KY749908	Ecuador:
	Pteronymia hara hara		E-39-43	DQ157538	DQ177983		Ecuador: Sucumbios, La Bonita
	Pteronymia hara hara		21135	KY750065	KY750150	KY749909	Ecuador: Zamora-Chinchipe, San Francisco
	Pteronymia hara hara		ME10-371	KY750218		KY749910	Ecuador: Napo, Cocodrilo

Pteronymia hara sspnov 5	LEP-04484	KY750019			Ecuador: West Pichincha, km 20 Pacto-Guayabillas rd.
Pteronymia hara sspnov 5	LEP-04485	KY750020			Ecuador: West Pichincha, 12 km SW Las Tolas
Pteronymia inania inania	20070	KY750067		KY749913	Ecuador:
Pteronymia inania inania	ME10-178	JX573818	JX573745	KY749911	Ecuador: Napo, Cocodrilo
Pteronymia inania inania	ME10-385	KY750066		KY749912	Ecuador: Napo, Cocodrilo
Pteronymia latilla barilla	20069	KY750068		KY749914	Ecuador:
Pteronymia latilla fulvescens	04-SRNP-48070	KY750069	KY750151		Costa Rica: Guanacaste, Area de Conservacion Guanacaste, Sector Cacao, Quebrada Otilio
Pteronymia latilla fulvescens	04-SRNP-48094	KY750112			Costa Rica: Guanacaste, Area de Conservacion Guanacaste, Sector Cacao, Quebrada Otilio
Pteronymia latilla nigricans	LEP-11341	KY750018			Colombia: Antioquia
Pteronymia latilla nigricans	PT007	KY750070	KY750152	KY749915	Colombia: Antioquia Amagá - "EL Socorro - F2"
Pteronymia latilla nigricans	PT008	KY750071	KY750153	KY749916	Colombia: Antioquia Fredonia - "La Sierra - F2"
Pteronymia laura	HW-6	KY750221			Colombia: Cundinamarca, Gruadas
Pteronymia lonera	2983	KY750072	KY750154	KY749917	Panama: Fortuna, Continental Divide Trail
Pteronymia lonera	8327	KY750073	KY750155	KY749918	Panama: Fortuna, Quebrada Hornito Trail
Pteronymia medellina	BMC-2203	KY750074		KY749919	Colombia: Cauca, Cajibío, Bellavista
Pteronymia medellina	PT005	KY750217			Colombia: Antioquia Ciudad Bolivar - "Las Acacias- F1"
Pteronymia obscuratus	8978	KY750075	KY750156	KY749920	Panama: Rio Pi-as, Campsite
Pteronymia olimba	MJ07-654	KY750120			Peru: CU San Pedro
Pteronymia olimba	MJ07-655	KY750017			Bolivia: LP, Caranavi
Pteronymia oneida oneida	20036	KY750076	KY750157	KY749921	Ecuador: Zamora-Chinchipe, San Francisco, casa de Arcoiris , km 23 Loja-Zamora rd.
Pteronymia oneida oneida	20037	KY750077	KY750158	KY749922	Ecuador: Zamora-Chinchipe, San Francisco, casa de Arcoiris , km 23 Loja-Zamora rd.
Pteronymia oneida oneida	21304	JX573819	JX573746	KY749923	Ecuador: Zamora-Chinchipe, San Francisco
Pteronymia oneida oneida	21360	KY750078	KY750159	KY749924	Ecuador: Zamora-Chinchipe, San Francisco
Pteronymia oneida oneida	E-43-1	DQ157539	DQ177984		Ecuador: Sucumbios, La Bonita - Tulcan Road
Pteronymia oneida oneida	ME10-347	KY750079		KY749925	Ecuador: Napo, Yanayacu
Pteronymia ozia ozia	20049	KY750080	KY750160		Ecuador: Morona-Santiago, Yakunk-Cutucu trail, lower ridge
Pteronymia ozia ozia	20050	KY750004	KY750161	KY749926	Ecuador: Morona-Santiago, Loma Kilamo , Macas
Pteronymia ozia ozia	21024	JX573820	JX573747	KY749927	Ecuador: Zamora-Chinchipe, San Francisco
Pteronymia ozia browni	ME10-420	KY750121		KY749928	Ecuador: Napo, Cocodrilo
Pteronymia ozia tanampaya	21599	KY750081	KY750162	KY749929	Peru: CU, San Pedro
Pteronymia parva	00-SRNP-1047	GU334307			Costa Rica: Alajuela, Area de Conservacion Guanacaste, Sector San Cristobal, Melina Bufalo
Pteronymia parva	00-SRNP-1052	GU334310			Costa Rica: Alajuela, Area de Conservacion Guanacaste, Sector San Cristobal, Melina Bufalo
Pteronymia parva	08-SRNP-476	JQ537070	KY750163	KY749930	Costa Rica:
Pteronymia picta notilla	07-SRNP-36576	JQ536861			Costa Rica:
Pteronymia picta notilla	08-SRNP-4914	JQ538429			Costa Rica: Area de Conservacion Guanacaste, Sector San Cristobal, Sendero Carmona
Pteronymia picta notilla	11-SRNP-30301	JQ526880			Costa Rica:
Pteronymia picta notilla	04-SRNP-2817	GU334313	KY750164	KY749931	Costa Rica: Alajuela, Colonia Bolanos, Sector San Cristobal, Vado Rio Cucaracho
Pteronymia picta notilla	04-SRNP-45731	GU334311	KY750165	KY749932	Costa Rica: Guanacaste, Area de Conservacion Guanacaste, Sector Cacao, Quebrada Heliconia
Pteronymia picta notilla	8342	KY750082	KY750166	KY749933	Panama: Fortuna, Quebrada Hornito Trail
Pteronymia picta notilla	8846	KY750083	KY750167	KY749934	Panama: Darien, Cana Cerro Pirre
Pteronymia picta notilla	8847	KY750084	KY750168	KY749935	Panama: Darien, Cana Cerro Pirre
Pteronymia picta picta	PT013	KY750216	KY750169	KY749936	Colombia: Antioquia Amalfi "Porce -La Hacienda"
Pteronymia picta picta	PT016	KY750085	KY750170	KY749937	Colombia: Antioquia Amalfi "Porce -La Hacienda"
Pteronymia picta picta	PT019	KY750086	KY750171	KY749938	Colombia: Antioquia Amalfi "Porce -La Hacienda"
Pteronymia primula primula	306	KY750087	KY750172	KY749939	Ecuador: Azuay, Communidad Shuar Mirador, 70 km E of Macas (Macas to Puyo road)
Pteronymia primula primula	20213	EU069103	EU069250	KY749940	Ecuador: Orellana, Rio Anangu
Pteronymia primula primula	20255	EU069104	EU069251	KY749941	Ecuador: Orellana, Rio Anangu
Pteronymia primula primula	20755	EU068973	EU069253	KY749942	Ecuador: Orellana, Rio Anangu
Pteronymia primula primula	05-920	EU068942	KY750173	KY749943	Peru: San Martin, PNCAZ, Quebrada Machaco, Cachatigre, Puesto de Control 32
Pteronymia rufocincta	MJ07-659	KY750015			Mexico: Michoacán, La Nuez
Pteronymia rufocincta	MJ07-660	KY750016			Mexico: Michoacán, La Nuez
Pteronymia sao antisao	04-169	KY750088	KY750174	KY749944	Peru: Ucayali, Pucallpa, Lago Yarinacocha, Caño Tushmo
Pteronymia sao antisao	04-170	KY750089	KY750175	KY749945	Peru: Ucayali, Pucallpa, Lago Yarinacocha, Caño Tushmo
Pteronymia sao antisao	02-560	EU068943	KY750177	KY749947	Peru: San Martin, Convento
Pteronymia sao sspnov 1	20286	EU069105	EU069255	KY749948	Ecuador: Orellana, Rio Anangu
Pteronymia sao sspnov 1	20520	EU068945	KY750178	KY749949	Ecuador: Orellana, Rio Anangu
Pteronymia sao sspnov 1	20522	EU068946	KY750179	KY749950	Ecuador: Orellana, Rio Anangu
Pteronymia sao sspnov 1	20045	KY750090	KY750176	KY749946	Ecuador: Napo, Rio Yuturi, lodge trail
Pteronymia serrata amplificata	20033	KY750091	KY750180	KY749951	Ecuador: Zamora-Chinchipe, San Francisco, casa de Arcoiris , km 23 Loja-Zamora rd.
Pteronymia serrata amplificata	21139	JX573821	JX573748	KY749952	Ecuador: Zamora-Chinchipe, San Francisco

	Pteronymia serrata amplificata		21516	KY750092	KY750181	KY749953	Ecuador: Zamora-Chinchipe, San Francisco
	Pteronymia sexpunctata sexpunctata		MJ07-663	KY750013			Peru: Amazonas, Quebrada Gebil
	Pteronymia sexpunctata sspnov 1		MJ07-665	KY750122		KY749954	Peru: Cuzco, San Pedro
	Pteronymia sexpunctata sspnov 1		MJ07-666	KY750014			Peru: Cuzco, San Pedro
	Pteronymia simplex simplex		8407	KY750093	KY750182	KY749955	Panama: Fortuna, Cafe Vista Hermosa
	Pteronymia tamina		02-802		KY750202	KY749984	Peru: San Martín, Puente Serranoyacu
	Pteronymia tamina		21474	JX573822	JX573749	KY749956	Ecuador: Zamora-Chinchipe, San Francisco
	Pteronymia tamina		21479	KY750094	KY750183	KY749957	Ecuador: Zamora-Chinchipe, San Francisco
	Pteronymia teresita teresita		LEP-04482	KY750012			Ecuador: West Pichincha, 12 km SW Las Tolas
	Pteronymia teresita teresita		LEP-04483	KY750011			Ecuador: West Pichincha, 7 km SW Las Tolas
	Pteronymia ticida sspnov 1		20861	KY750001	KY750191	KY749968	Ecuador: Orellana, Rio Anangu - DEFINITELY MISLABELLED
	Pteronymia ticida sspnov 3		05-1035	KY750010			Peru: San Martin, Venceremos + 4 km (Segundo Baden)
	Pteronymia ticida sspnov 3		05-1163		KY750203	KY749985	Peru: San Martin, Venceremos + 4 km (Segundo Baden)
	Pteronymia ticida ticida		21263	JX573824	JX573751	KY749967	Ecuador: Zamora-Chinchipe, San Francisco
	Pteronymia ticida ticida		20077	KY750098	KY750188	KY749964	Ecuador:
	Pteronymia ticida ticida		20078	KY750099	KY750189	KY749965	Ecuador:
	Pteronymia ticida ticida		20079	KY750100	KY750190	KY749966	Ecuador:
	Pteronymia ticida yungava		21592	KY750101	KY750192	KY749969	Peru: CU, San Pedro
	Pteronymia thabena denticulata	*teresita*	02-797	KY750095	KY750185	KY749959	Peru: San Martín, Puente Serranoyacu
	Pteronymia thabena denticulata	*teresita*	02-942	KY750096	KY750186	KY749960	Peru: San Martín, Puente Serranoyacu
	Pteronymia thabena denticulata	*teresita*	21606	KY750213	KY750184	KY749958	Peru: CU, San Pedro
	Pteronymia thabena thabena	*teresita*	20057	KY750215			Ecuador: Morona-Santiago, km 14 Lima-Gualaceo rd.,
	Pteronymia thabena thabena	*teresita*	21311	KY750097	KY750187	KY749961	Ecuador: Zamora-Chinchipe, San Francisco
	Pteronymia thabena thabena	*teresita*	21480	JX573823	JX573750	KY749962	Ecuador: Zamora-Chinchipe, San Francisco
	Pteronymia thabena thabena	*teresita*	ME10-386	KY750116		KY749963	Ecuador: Napo, Cocodrilo
	Pteronymia tucuna		02-3168	KY750102	KY750193	KY749970	Peru: Cuzco, Palma Real
	Pteronymia tucuna		05-1372	KY750103	KY750194	KY749971	Peru: San Martin, Boca Toma Rio Shilcayo
	Pteronymia tucuna		PE-10-12	DQ157542	DQ177987		Peru: Cuzco, Quebrada Chaupimayo
	Pteronymia veia_EAST linzera	*veia*	20081	KY750104	KY750195	KY749972	Ecuador:
	Pteronymia veia_EAST linzera	*veia*	20083	KY750214			Ecuador: Loja, Yangana, Rio ChiriguaÃ±a
	Pteronymia veia_EAST linzera	*veia*	E-43-16	DQ069242.1	DQ073036.1	AY848736	Ecuador: Sucumbios, La Bonita - Tulcan Road
	Pteronymia veia_EAST linzera	*veia*	ME10-453	KY750105			Ecuador: Napo, Cocodrilo
	Pteronymia veia_WEST sspnov 2	*veia*	LEP-04480	KY750009			Ecuador: West Pichincha, Bellavista Lodge ridge road
	Pteronymia veia_WEST sspnov 2	*veia*	LEP-06893	KY750008			Ecuador: West Carchi, East of Maldonado
	Pteronymia vestilla sparsa		20270	EU069106	EU069256	KY749975	Ecuador: Orellana, Rio Anangu
	Pteronymia vestilla sparsa		20756	EU069107	EU069257	KY749976	Ecuador: Orellana, Rio Anangu
	Pteronymia vestilla sspnov 2		04-469	KY750107	KY750197	KY749974	Peru: San Martín, Km22 Nuevo Lima - Selva Andina
	Pteronymia vestilla sspnov 2		05-267	KY750106	KY750196	KY749973	Peru: Loreto, PNCAZ, Pongo del Río Pauya
	Pteronymia vestilla sspnov 2		04-463	EU068948	KY750198	KY749977	Peru: San Martín, Km22 Nuevo Lima - Selva Andina
	Pteronymia vestilla sspnov 2		LdeS11-610	KY750002	KY750199	KY749978	Peru: San Martin, Bocatoma Rio Shilcayo
	Pteronymia vestilla ucaya		BAKU12	KY750111		KY749979	Brazil: Acre, Marechal Thaumaturgo, Res. Extr. Alto Rio Jurua, Colocaçao Pedra Pintada
	Pteronymia zerlina pronuba		LEP-08685	KY750006			Ecuador: West Carchi, East of Maldonado, Río Chorro Blanco
	Pteronymia zerlina pronuba		LEP-08686	KY750007			Ecuador: West Pichincha, km 9 Pacto-Guayabillas rd.
	Pteronymia spnov 1		HW-5	KY750048		KY749887	Colombia: Cundinamarca, Santandrecito
	Pteronymia spnov 2		164	KY750049	KY750136	KY749888	Ecuador: Loja, Vilcabamba Ecolodge, Eastern Trail from Ruinas Lodge to Rumi Wilco
	Pteronymia spnov 2		21064	KY750050		KY749889	Ecuador: Zamora-Chinchipe, San Francisco
	Pteronymia spnov 2		21336	KY750051	KY750137	KY749890	Ecuador: Zamora-Chinchipe, San Francisco
	Pteronymia spnov 3		20071	KY750053		KY749893	Ecuador:
	Pteronymia spnov 3		ME10-322	JX573816	JX573743	KY749892	Ecuador: Napo, Yanayacu
	Pteronymia spnov 4		MJ07-639	KY750022			Panama: Darien, Cana Cerro Pirre
**Outgroups**							
Ithomiini	*Aeria eurimedia*		BAKU62	KY750225	KY750208		
Ithomiini	*Aremfoxia ferra ferra*		G104	KY750223			
Ithomiini	*Athesis acrisione acrisione*		LEP-06463	KY750220	KY750206		
Ithomiini	*Athyrtis mechanitis*		RB359	DQ069230	DQ073022		
Ithomiini	*Brevioleria seba oculata*		20458	EU069025	EU069120	KY749998	
Ithomiini	*Callithomia lenea zelie*		20233	EU069032	EU069126	KY749989	
Ithomiini	*Ceratinia tutia poecila*		20216	KY750113	KY750209	KY749991	
Ithomiini	*Dircena olyras sspnov*		8279			KY749999	

Ithomiini	*Dircenna loreta melini*	04-364	JX573758	JX573687	KY749992
Ithomiini	*Elzunia humboldt cassandrina*	LEP-06870	KF268430	KF268427	KY749986
Ithomiini	*Episcada carcinia*	BAKU17	KY750003	KY750211	
Ithomiini	*Epityches eupompe*	B12-2	DQ157492	DQ177938	
Ithomiini	*Eutresis hypereia imitatrix*	ME10-458	JX573762	JX573691	
Ithomiini	*Forbestra olivencia juntana*	20325	EU069038	EU069139	KY749995
Ithomiini	*Godyris hewitsonii sspnov*	21050	JX573764	JX573693	KX362244
Ithomiini	*Greta andromica andania*	21054	JX573767	JX573696	KX362147
Ithomiini	*Haenschia derama*	G109	KY750114		KY749987
Ithomiini	*Heterosais nephele nephele*	20667	EU069043	EU069148	KX362212
Ithomiini	*Hyalenna pascua*	BAKU4	KY750115	KY750210	KY749990
Ithomiini	*Hyalyris antea*	E-30-4	DQ069237	DQ073029	KY749988
Ithomiini	*Hypoleria xenophis*	05-624	KX362050	KX361952	KX362215
Ithomiini	*Hyposcada illinissa napoensis*	21750	EU069053	EU069163	FN568131
Ithomiini	*Hypothyris anastasia honesta*	20507	EU068811	EU069164	KY749994
Ithomiini	*Ithomia agnosia agnosia*	8895	AY713040	AY704014	AY704060
Ithomiini	*Mcclungia cymo*	ME11-159	JX573792	JX573720	KX362136
Ithomiini	*Mechanitis mazaeus mazaeus*	20781	EU069074	EU069195	FJ446071
Ithomiini	*Megoleria orestilla orestilla*	05-1028	FN646248	FN551047	FN568149
Ithomiini	*Melinaea menophilus hicetas*	02-1262	DQ078434	KY750212	HM052088
Ithomiini	*Methona confusa*	BAKU59	KY750222	KY750205	KY749993
Ithomiini	*Napeogenes pharo pharo*	20226	EU069089	EU069225	FJ469107
Ithomiini	*Oleria rubescens*	8369	DQ085460	DQ085455	AY848733
Ithomiini	*Olyras crathis montagui*	ME10-415	JX573806	JX573733	KY749997
Ithomiini	*Pachacutia mantura*	MC11-63	KX362127		KX362197
Ithomiini	*Pagyris cymothoe sylvella*	8946	AY713038	AY704012	KY749996
Ithomiini	*Paititia neglecta*	02-1244	DQ073038	DQ073034	AY848735
Ithomiini	*Patricia dercyllidas*	E35-7	DQ157531	DQ177976	
Ithomiini	*Placidina euryanassa*	B16-1	DQ157532	DQ177977	
Ithomiini	*Pseudoscada florula aureola*	20214	EU068971	EU069246	HM052165
Ithomiini	*Scada zibia batesi*	20236	EU069109	EU069260	
Ithomiini	*Thyridia psidii ino*	20354	EU069110	EU069262	
Ithomiini	*Tithorea tarricina pinthias*	06-SRNP-2593	KY750224	KY750207	
Ithomiini	*Veladyris pardalis aurea*	ME10-38	KX362030	KX361955	KX362141
Ithomiini	*Velamysta pupilla sspnov*	424	KX362082	KX362004	KX362234
Danaini	*Amauris ellioti*	NW86-5	AY218234	AY218253	
Danaini	*Anetia briarea*	NW152-6	EU141366	EU136673	
Danaini	*Danaus plexippus*	NW108 21	DQ018954	DQ018921	
Danaini	*Euploea camaralzeman*	NW70-8	AY090205	AY090171	
Danaini	*Idea leuconoe*	NW84-14	GQ864778	GQ864872	
Danaini	*Lycorea halia*	NW122-19	GQ864788	GQ864882	
Danaini	*Lycorea ilione*	B17-37	GQ864780	GQ864874	
Danaini	*Parantica aspasia*	NW112-8	GQ864799	GQ864893	
Danaini	*Parantica luzonensis*	NW118-17	GQ864779	GQ864873	
Tellervini	*Tellervo zoilus*	QL5	GQ864812	GQ864906	

Supplementary table S2. Morphological character data matrix.

	1	2	3	4	5	6	7	8	9	10	11	12	13	14	15	16	17	18	19	20	21	22	23	24	25	26	27	28	29	30	31	32	33	34	35	36	37	38	39	40	41	42	43	44	45	46	47	48	49	50	51	52	53	54	55	56	57	58	59	60	61	62	63	64	65	66	67	68	69	70	71	72	73	74	75	76	77	78	79	80	81	82	83	84	85	86	87
Episcada_apuleia	0	0	0	0	0	-	0	0	0	0	0	0	0	0	0	0	1	1	1	0	-	0	0	0	0	0	1	0	-	2	0	1	0	1	1	0	1	1	0	0	0	0	0	0	0	1	0	0	0	1	-	-	-	0	-	-	0	0	1	2	0	0	-	0	-	0	-	0	0	-	0	0	1	1	0	1	1	0	1	0	1	1	1	0	0	0	0
Dircenna_adina	0	0	0	0	0	0	0	0	0	0	0	0	0	0	0	0	0	0	0	0	-	0	0	0	0	0	0	0	-	0	0	0	0	0	0	0	0	0	0	0	0	0	0	0	0	0	-	0	0	0	0	-	-	0	-	-	0	0	0	-	0	0	-	0	-	0	-	0	0	-	0	0	0	0	0	0	0	0	0	0	0	0	0	0	0	0	0	0
Pteronymia_latilla	0	0	0	1	0	0	0	0	0	0	1	0	1	-	-	0	1	0	0	0	-	1	0	-	0	0	0	1	-	1	1	1	0	0	1	3	5	0	0	0	0	1	?	0	0	1	3	?	0	0	1	0	0	1	0	0	1	0	1	0	?	1	1	1	2	0	-	0	1	1	?	0	2	1	1	1	1	0	1	0	2	?	1	0	?	0	1
Pteronymia_alcmena	0	0	0	1	0	0	0	0	0	0	0	0	0	0	4	0	1	0	0	0	-	1	1	1	0	0	0	1	-	1	1	1	0	1	1	0	A	0	0	0	0	1	0	0	1	?	?	?	?	?	?	?	?	?	?	?	?	?	?	?	?	?	?	?	?	?	?	?	?	?	?	?	?	?	?	?	?	?	?	?	?	?	?	?	?	?	?	?
Pteronymia_aletta	0	0	0	0	0	0	0	0	0	2	0	2	0	0	2	0	1	0	0	1	0	1	0	1	0	0	1	1	-	1	1	1	0	1	1	0	7	1	0	0	0	0	1	0	0	1	3	1	0	0	1	0	0	1	0	0	1	0	1	0	1	1	1	1	2	0	-	0	1	0	0	0	2	1	?	1	1	0	1	0	2	?	?	0	0	0	0
Pteronymia_alicia	0	0	0	0	0	0	1	1	1	0	0	1	0	0	2	0	1	0	0	1	0	1	0	1	0	0	0	2	1	1	1	1	0	1	0	?	?	?	?	?	?	?	?	?	1	?	?	?	?	?	?	?	?	?	?	?	?	?	?	?	?	?	?	?	?	?	?	?	?	?	?	?	?	?	?	?	?	?	?	?	?	?	?	?	?	?	?	?
Pteronymia_alida	0	0	0	0	0	1	0	0	0	2	1	2	0	0	1	0	0	1	1	2	-	0	0	0	1	1	0	2	0	1	1	1	1	0	1	1	1	1	0	0	0	0	1	1	1	1	0	0	0	1	-	-	-	0	-	-	1	0	1	1	1	1	0	1	1	0	-	0	0	-	0	0	2	1	1	1	1	0	3	0	1	1	1	0	0	0	0
Pteronymia_alina	1	0	0	1	0	0	0	0	0	0	1	0	0	0	4	0	1	0	0	0	-	1	0	1	0	0	0	1	-	1	1	1	0	0	1	2	3	0	0	0	0	1	?	0	1	1	3	0	0	0	1	0	0	0	-	-	0	0	1	0	1	1	1	1	2	0	-	0	1	1	0	0	2	1	1	1	1	0	1	0	1	1	1	0	0	0	?
Pteronymia_alissa	0	0	0	1	0	0	0	0	0	1	0	2	0	0	?	0	1	0	0	0	-	1	1	1	0	0	0	1	-	1	1	1	0	1	1	3	A	0	?	0	0	0	?	0	1	?	?	?	?	?	?	?	?	?	?	?	?	?	?	?	?	?	?	?	?	?	?	?	?	?	?	?	?	?	?	?	?	?	?	?	?	?	?	?	?	?	?	0
Pteronymia_andreas	0	0	0	1	0	0	0	0	0	1	0	2	0	0	4	0	1	0	0	0	-	1	1	1	0	0	0	1	-	1	1	1	0	1	1	3	A	0	0	0	0	0	?	0	1	1	3	1	0	0	1	0	0	1	-	-	1	1	1	0	1	1	1	1	2	0	-	0	1	1	0	0	2	1	1	1	1	0	1	0	1	1	1	0	0	0	0
Pteronymia_artena	0	0	0	0	0	1	1	1	1	0	0	0	0	0	2	0	1	2	1	1	0	1	0	1	0	0	0	2	0	1	1	1	1	0	0	1	2	1	0	0	0	0	0	1	1	1	0	0	0	0	1	0	1	0	-	-	0	0	1	0	1	1	0	1	0	0	-	0	0	-	0	0	2	1	1	1	1	0	1	0	1	0	1	0	1	0	0
Pteronymia_asopo	1	0	0	1	0	0	0	0	0	0	1	0	0	0	4	0	1	0	0	0	-	1	0	1	0	0	0	1	-	1	1	1	0	0	1	?	?	0	?	?	?	?	?	0	1	?	?	?	?	?	?	?	?	?	?	?	?	?	?	?	?	?	?	?	?	?	?	?	?	?	?	?	?	?	?	?	?	?	?	?	?	?	?	?	?	?	?	?
Pteronymia_calgiria	0	0	0	1	0	0	0	0	0	0	0	0	0	0	4	0	1	0	0	0	-	1	0	1	0	0	0	1	-	1	1	1	0	0	1	3	5	0	0	0	0	1	?	0	0	?	?	?	?	?	?	?	?	?	?	?	?	?	?	?	?	?	?	?	?	?	?	?	?	?	?	?	?	?	?	?	?	?	?	?	?	?	?	?	?	?	?	1
Pteronymia_carlia	0	0	0	0	0	0	0	0	0	0	0	2	0	0	2	0	1	0	0	1	1	1	0	1	0	0	1	1	-	1	1	1	0	1	1	0	6	1	0	0	0	0	1	0	1	1	3	?	0	0	1	0	0	1	0	0	1	0	1	0	1	1	1	1	2	0	-	0	1	1	0	0	2	1	?	1	1	0	1	0	2	?	?	0	0	0	0
Pteronymia_cotytto	0	0	0	0	0	0	0	0	0	2	0	2	0	0	2	0	1	0	0	1	1	1	0	1	0	2	1	1	-	1	1	1	0	1	1	0	7	1	0	0	0	0	1	0	1	?	?	?	?	?	?	?	?	?	?	?	?	?	?	?	?	?	?	?	?	?	?	?	?	?	?	?	?	?	?	?	?	?	?	?	?	?	?	?	?	?	?	0
Pteronymia_donella	0	1	0	0	0	1	0	0	0	2	1	2	0	0	2	0	1	0	0	0	-	1	0	1	0	0	0	1	-	1	0	0	0	0	1	1	1	1	0	0	0	0	0	1	0	?	?	?	?	?	?	?	?	?	?	?	?	?	?	?	?	?	?	?	?	?	?	?	?	?	?	?	?	?	?	?	?	?	?	?	?	?	?	?	?	?	?	0
Pteronymia_euritea	0	0	0	0	0	0	0	0	0	0	0	2	0	0	2	0	1	0	0	1	0	1	0	1	0	0	0	1	-	2	1	1	0	0	1	0	8	1	0	0	0	0	1	0	0	1	0	1	0	0	1	0	0	1	0	1	0	0	1	0	1	1	1	1	2	1	1	0	1	0	0	0	2	1	?	1	1	0	1	0	1	?	?	0	?	0	0
Pteronymia_forsteri	0	0	0	0	0	1	0	0	0	0	1	2	0	0	3	0	1	1	1	0	-	1	0	1	0	0	0	2	1	1	0	0	0	1	0	1	1	1	0	0	0	0	1	1	1	?	?	?	?	?	?	?	?	?	?	?	?	?	?	?	?	?	?	?	?	?	?	?	?	?	?	?	?	?	?	?	?	?	?	?	?	?	?	?	?	?	?	0
Pteronymia_fulvimargo	1	0	0	1	0	0	0	0	0	0	1	0	0	1	4	0	1	0	0	0	-	1	0	1	0	0	0	1	-	1	1	1	0	0	1	2	3	0	?	0	?	1	?	0	1	?	?	?	?	?	?	?	?	?	?	?	?	?	?	?	?	?	?	?	?	?	?	?	?	?	?	?	?	?	?	?	?	?	?	?	?	?	?	?	?	?	?	?
Pteronymia_fumida	0	0	0	0	0	0	0	0	0	2	0	0	0	0	4	0	1	1	0	3	-	1	0	1	0	0	1	2	0	1	1	1	0	1	1	1	2	1	0	0	1	0	0	1	0	?	?	?	?	?	?	?	?	?	?	?	?	?	?	?	?	?	?	?	?	?	?	?	?	?	?	?	?	?	?	?	?	?	?	?	?	?	?	?	?	?	?	0
Pteronymia_gertschi	0	0	0	1	0	0	0	0	0	0	0	0	0	0	4	0	1	0	0	0	-	1	1	1	0	0	0	1	-	?	?	?	?	?	?	?	?	?	?	?	?	?	?	?	0	?	?	?	?	?	?	?	?	?	?	?	?	?	?	?	?	?	?	?	?	?	?	?	?	?	?	?	?	?	?	?	?	?	?	?	?	?	?	?	?	?	?	?
Pteronymia_glauca	0	0	0	0	0	0	0	0	0	0	0	2	0	0	4	0	1	0	0	1	0	1	0	1	0	0	1	1	-	2	1	1	0	0	1	0	9	1	0	0	0	0	0	0	1	?	?	?	?	?	?	?	?	?	?	?	?	?	?	?	?	?	?	?	?	?	?	?	?	?	?	?	?	?	?	?	?	?	?	?	?	?	?	?	?	?	?	?
Pteronymia_granica	0	0	0	0	0	0	0	0	0	2	0	1	0	0	5	0	1	0	1	1	0	1	0	1	1	1	0	2	0	1	1	1	1	1	0	1	1	1	0	0	1	0	0	1	1	?	?	?	?	?	?	?	?	?	?	?	?	?	?	?	?	?	?	?	?	?	?	?	?	?	?	?	?	?	?	?	?	?	?	?	?	?	?	?	?	?	?	0
Pteronymia_hara	0	1	0	0	0	1	0	0	0	2	1	1	0	0	5	0	0	1	1	[01]	-	1	0	1	0	3	1	2	0	1	1	1	1	1	0	1	1	1	0	0	1	0	0	1	1	?	?	?	?	?	?	?	?	?	?	?	?	?	?	?	?	?	?	?	?	?	?	?	?	?	?	?	?	?	?	?	?	?	?	?	?	?	?	?	?	?	?	0
Pteronymia_inania	0	0	0	0	0	1	0	0	0	2	1	2	0	0	1	0	0	1	1	2	-	0	0	0	1	1	1	1	-	1	1	1	1	0	1	1	1	1	0	0	0	0	1	1	1	1	1	0	0	0	1	1	-	1	1	-	0	0	0	-	0	1	1	1	2	0	-	0	1	0	1	1	-	1	0	2	2	1	2	1	1	0	0	1	1	1	0
Pteronymia_laura	0	0	0	1	0	0	0	0	0	0	0	0	0	0	4	0	1	0	0	0	-	1	1	1	0	0	0	1	-	1	1	1	0	1	1	3	A	0	0	0	0	1	?	0	1	?	?	?	?	?	?	?	?	?	?	?	?	?	?	?	?	?	?	?	?	?	?	?	?	?	?	?	?	?	?	?	?	?	?	?	?	?	?	?	?	?	?	0
Pteronymia_lonera	0	0	0	0	0	1	0	0	0	2	1	2	0	0	1	0	0	1	1	2	-	0	0	0	1	1	1	1	-	1	1	1	1	0	1	1	1	1	0	0	0	0	1	1	0	1	1	0	0	0	1	1	-	1	1	-	0	0	-	-	0	1	1	1	2	0	-	0	1	0	1	1	-	1	0	2	2	1	2	1	1	0	?	1	1	1	0
Pteronymia_medellina	0	0	0	1	0	0	0	0	0	1	0	0	0	0	4	0	1	0	0	1	0	1	0	1	0	0	1	1	-	1	1	1	0	1	1	0	9	1	0	0	0	0	0	0	1	1	3	1	0	0	1	0	0	0	-	0	1	0	1	0	1	1	1	1	2	0	-	0	1	1	0	0	?	1	?	1	1	0	3	0	1	?	?	0	?	0	0
Pteronymia_obscuratus	0	0	0	1	1	0	0	0	0	1	0	2	1	-	-	1	1	0	0	0	-	0	0	-	0	0	0	2	1	2	1	1	0	1	1	3	5	0	0	0	0	1	?	0	0	?	?	?	?	?	?	?	?	?	?	?	?	?	?	?	?	?	?	?	?	?	?	?	?	?	?	?	?	?	?	?	?	?	?	?	?	?	?	?	?	?	?	1
Pteronymia_olimba	0	0	1	0	0	0	0	0	0	0	0	0	0	0	2	0	1	2	1	1	0	1	0	1	0	0	1	2	0	1	1	1	1	1	0	?	?	?	?	?	?	?	?	?	0	?	?	?	?	?	?	?	?	?	?	?	?	?	?	?	?	?	?	?	?	?	?	?	?	?	?	?	?	?	?	?	?	?	?	?	?	?	?	?	?	?	?	?
Pteronymia_oneida	1	0	0	1	0	0	0	0	0	0	1	0	0	0	4	0	1	0	0	0	-	1	0	1	0	0	0	1	-	1	1	1	0	0	1	2	3	0	0	1	0	1	?	0	1	1	3	0	0	0	1	0	0	0	-	-	0	0	1	0	1	1	0	1	2	0	-	0	1	1	0	0	2	1	1	1	1	0	1	0	1	1	1	0	1	0	0
Pteronymia_ozia	1	0	0	1	0	0	0	0	0	0	1	0	0	0	4	0	1	0	0	0	-	1	0	1	0	0	0	1	-	1	1	1	0	0	1	2	3	0	0	1	0	1	?	0	1	1	3	0	0	0	1	0	0	0	-	-	0	0	1	0	1	1	1	1	2	0	-	0	1	1	0	0	2	1	1	1	1	0	1	0	1	1	1	0	1	0	?
Pteronymia_parva	0	0	0	0	0	0	0	0	0	2	0	2	0	0	2	0	1	2	0	1	0	1	0	1	0	0	1	1	-	1	1	1	0	1	1	0	7	1	0	0	0	0	1	0	0	1	0	0	0	0	1	0	0	1	0	1	0	0	1	0	1	1	1	1	2	1	1	0	1	0	0	?	?	?	?	?	?	?	?	?	?	?	?	?	?	?	?
Pteronymia_peteri	0	0	0	?	0	?	0	?	?	2	0	0	0	0	4	?	1	0	?	0	-	1	0	?	0	0	1	?	?	?	?	?	?	?	?	?	?	?	?	?	?	?	?	?	1	?	?	?	?	?	?	?	?	?	?	?	?	?	?	?	?	?	?	?	?	?	?	?	?	?	?	?	?	?	?	?	?	?	?	?	?	?	?	?	?	?	0	?
Pteronymia_picta	0	0	0	1	0	0	0	0	0	1	0	2	0	0	4	0	1	0	0	0	-	1	0	1	0	0	0	1	-	1	1	1	0	0	1	2	B	1	0	0	0	1	?	0	1	1	0	1	0	0	1	0	0	1	0	0	1	0	1	0	1	1	1	1	2	0	-	0	1	0	0	0	2	1	1	1	1	0	1	0	1	1	1	0	1	0	0
Pteronymia_primula	0	0	0	0	0	0	0	0	0	0	0	0	0	0	2	0	1	0	0	1	0	1	0	1	0	0	1	1	-	1	1	1	0	0	1	0	7	1	0	0	0	0	1	0	0	1	3	1	0	0	1	0	0	1	0	0	0	0	1	0	1	1	1	1	2	1	0	0	1	1	0	0	2	1	0	1	1	0	1	0	2	1	1	0	0	0	0
Pteronymia_rufocincta	0	0	0	0	0	0	0	0	0	2	0	2	0	0	2	0	1	0	0	1	0	1	0	1	0	2	1	1	-	1	1	1	0	1	1	0	7	1	0	0	0	0	1	0	1	?	?	?	?	?	?	?	?	?	?	?	?	?	?	?	?	?	?	?	?	?	?	?	?	?	?	?	?	?	?	?	?	?	?	?	?	?	?	?	?	?	?	0
Pteronymia_sao	0	0	0	1	1	0	0	0	0	1	0	2	1	-	-	1	1	0	0	0	-	0	0	-	0	0	0	2	1	2	1	1	0	1	1	3	5	0	0	0	0	1	?	0	0	1	2	0	1	0	1	0	0	1	0	0	0	0	1	0	0	1	0	1	1	0	-	1	1	2	0	0	2	1	0	1	1	0	1	0	1	1	1	0	1	0	1
Pteronymia_serrata	0	0	0	0	0	1	0	0	0	2	1	2	0	0	1	0	0	1	1	2	-	0	0	1	1	1	0	2	0	2	1	1	1	0	1	1	1	1	0	0	0	0	1	1	0	1	0	0	0	0	1	0	1	0	-	-	0	0	1	1	0	1	0	1	0	0	-	0	0	-	0	?	?	1	?	1	?	?	?	0	?	?	1	0	0	0	0
Pteronymia_sexpunctata	0	0	0	1	0	0	0	0	0	0	0	0	0	0	4	0	1	0	0	0	-	1	1	1	0	0	0	1	-	1	1	1	0	0	1	2	4	1	1	0	0	1	?	0	0	?	?	?	?	?	?	?	?	?	?	?	?	?	?	?	?	?	?	?	?	?	?	?	?	?	?	?	?	?	?	?	?	?	?	?	?	?	?	?	?	?	?	?
Pteronymia_simplex	0	0	0	1	0	0	0	0	0	1	0	2	0	0	3	0	1	0	0	0	-	1	0	1	0	0	1	1	-	1	1	1	0	1	1	3	B	0	1	0	0	1	?	0	1	?	?	?	?	?	?	?	?	?	?	?	?	?	?	?	?	?	?	?	?	?	?	?	?	?	?	?	?	?	?	?	?	?	?	?	?	?	?	?	?	?	?	1
Pteronymia_tamina	1	0	0	1	0	0	0	0	0	0	1	0	0	0	4	0	1	0	0	0	-	1	0	1	0	0	0	1	-	1	1	1	0	0	1	2	3	0	0	1	0	1	?	0	1	?	?	?	?	?	?	?	?	?	?	?	?	?	?	?	?	?	?	?	?	?	?	?	?	?	?	?	?	?	?	?	?	?	?	?	?	?	?	?	?	?	?	?
Pteronymia_teresita	0	0	0	0	0	1	0	0	0	2	0	2	0	0	1	0	0	1	1	2	-	0	0	0	1	1	1	1	-	1	1	1	1	0	1	1	1	1	?	0	0	0	1	1	1	?	?	?	?	?	?	?	?	?	?	?	?	?	?	?	?	?	?	?	?	?	?	?	?	?	?	?	?	?	?	?	?	?	?	?	?	?	?	?	?	?	?	0
Pteronymia_thabena	0	0	0	0	0	1	0	0	0	2	1	2	0	0	1	0	0	1	1	2	-	0	0	0	1	1	1	1	-	1	1	1	1	0	1	1	1	1	0	0	0	0	1	1	1	1	1	0	0	0	1	1	-	1	1	-	0	0	0	-	0	1	1	1	2	0	-	0	1	0	1	1	-	1	0	2	2	1	2	1	1	0	0	1	1	1	0
Pteronymia_ticida	1	0	0	1	0	0	0	0	0	0	1	0	0	0	4	0	1	0	0	0	-	1	0	1	0	0	0	1	-	1	1	1	0	0	1	2	3	0	0	1	0	1	?	0	1	1	3	1	0	0	1	0	0	1	0	1	0	0	1	0	1	1	1	1	2	0	-	0	0	-	0	0	2	1	1	1	1	0	1	0	1	1	1	0	1	0	?
Pteronymia_tucuna	0	0	0	1	0	0	0	0	0	0	1	0	1	-	-	0	1	0	0	0	-	1	0	-	0	0	0	1	-	1	1	1	0	1	1	3	5	0	?	0	?	1	?	0	0	?	?	?	?	?	?	?	?	?	?	?	?	?	?	?	?	?	?	?	?	?	?	?	?	?	?	?	?	?	?	?	?	?	?	?	?	?	?	?	?	?	?	1
Pteronymia_veiaEAST	1	0	0	1	0	0	0	0	0	0	1	0	0	0	4	0	1	0	0	0	-	1	0	1	0	0	0	1	-	1	1	1	0	0	1	2	3	0	0	1	0	1	?	0	1	1	3	1	0	0	1	0	0	1	0	1	0	0	1	0	1	1	0	1	0	0	-	0	1	?	0	0	?	1	?	1	1	0	1	0	1	?	?	0	?	0	?
Pteronymia_veiaWEST	1	0	0	1	0	0	0	0	0	0	1	0	0	0	4	0	1	0	0	0	-	1	0	1	0	0	0	1	-	1	1	1	0	0	1	2	3	0	?	0	?	1	?	0	1	?	?	?	?	?	?	?	?	?	?	?	?	?	?	?	?	?	?	?	?	?	?	?	?	?	?	?	?	?	?	?	?	?	?	?	?	?	?	?	?	?	?	0
Pteronymia_vestilla	0	0	0	0	0	0	0	0	0	0	0	0	0	0	2	0	1	2	0	1	0	1	0	1	0	0	1	2	0	1	1	1	0	1	1	0	7	1	0	0	0	0	1	0	0	1	0	1	0	0	1	0	0	1	0	0	1	0	1	0	1	1	1	1	2	0	-	0	1	2	0	0	2	1	0	1	1	0	1	0	2	1	1	0	0	0	0
Pteronymia_zerlina	1	0	0	1	0	0	0	0	0	0	1	0	0	0	4	0	1	0	0	0	-	1	0	1	0	0	0	1	-	1	1	1	0	0	1	2	3	0	?	0	?	1	?	0	1	?	?	?	?	?	?	?	?	?	?	?	?	?	?	?	?	?	?	?	?	?	?	?	?	?	?	?	?	?	?	?	?	?	?	?	?	?	?	?	?	?	?	0
Pteronymia_spnov1	0	0	0	1	0	0	0	0	0	0	0	2	0	0	4	0	1	0	0	1	0	1	0	1	0	0	1	2	1	1	1	1	0	1	1	2	9	1	0	0	0	0	?	0	1	?	?	?	?	?	?	?	?	?	?	?	?	?	?	?	?	?	?	?	?	?	?	?	?	?	?	?	?	?	?	?	?	?	?	?	?	?	?	?	?	?	?	?
Pteronymia_spnov2	1	0	0	1	0	0	0	0	0	0	1	0	0	0	4	0	1	0	0	0	-	1	0	1	0	0	0	1	-	1	1	1	0	0	1	2	3	0	0	1	0	1	?	0	1	1	3	0	0	0	1	0	0	0	-	-	1	0	1	0	1	1	0	1	2	0	-	-	1	1	0	0	2	1	1	1	1	0	1	0	1	1	1	0	1	0	1
Pteronymia_spnov3	0	0	0	0	0	1	0	0	0	2	1	2	0	0	1	0	0	1	1	2	-	0	0	0	1	1	0	2	0	1	1	1	0	0	1	1	1	1	0	0	0	0	1	1	1	1	0	0	0	1	-	-	-	0	-	-	0	0	1	1	0	1	0	1	0	0	-	0	0	-	0	0	2	1	1	1	1	0	3	0	1	1	1	0	0	0	0
Pteronymia_spnov4	0	0	0	0	0	0	0	0	0	0	0	2	0	0	2	0	1	0	0	1	0	1	0	1	0	0	0	1	-	2	1	1	-	-	1	0	8	1	0	0	0	0	1	0	0	?	?	?	?	?	?	?	?	?	?	?	?	?	?	?	?	?	?	?	?	?	?	?	?	?	?	?	?	?	?	?	?	?	?	?	?	?	?	?	?	?	?	?

Supplementary table S3. Partition Finder results for RAxML and MrBayes

	Partition	Best model
	CO1_pos2, EF1A_pos2	GTR+I+G
	CO2_pos3, CO1_3	GTR+I+G
RAxML	CO2_pos1, CO2_pos2, CO1_2	GTR+I+G
	tektin_pos1, tektin_pos2, tRNAleu	GTR+I+G
	EF1A_pos3, tektin_pos3	GTR+G
	EF1A_pos1	GTR+I+G
	CO1_pos2 EF1A_pos2	HKY+I+G
	CO2_pos3 CO1_pos3	GTR+I+G
	CO2_pos1 CO1_pos1	GTR+I+G
MrBayes	CO2_pos2 tektin_pos2 tRNAleu	GTR+I+G
	tektin_pos3	GTR+G
	tektin_pos1	GTR+I+G
	EF1A_pos1	GTR+I+G
	EF1A_pos3	SYM+G

Supplementary table S4 Partition Finder results for BEAST

Partition	Best model
CO2_pos1, CO2_pos2, CO1_pos1, NC, tRNAleu	GTR+I+G
CO1_pos2, EF1A_pos2	HKY+I+G
CO2_pos3, CO1_pos3	GTR+I+G
tektin_pos1, tektin_pos2	TrN+I+G
tektin_pos3	TrN+G
EF1A_pos1	TrN+I+G
EF1A_pos3	SYM+G

Supplementary figure S1: Phylogenetic tree of *Pteronymia* obtained with RAxML (sco.h-its.org/ exelixis/web/software/raxml/).

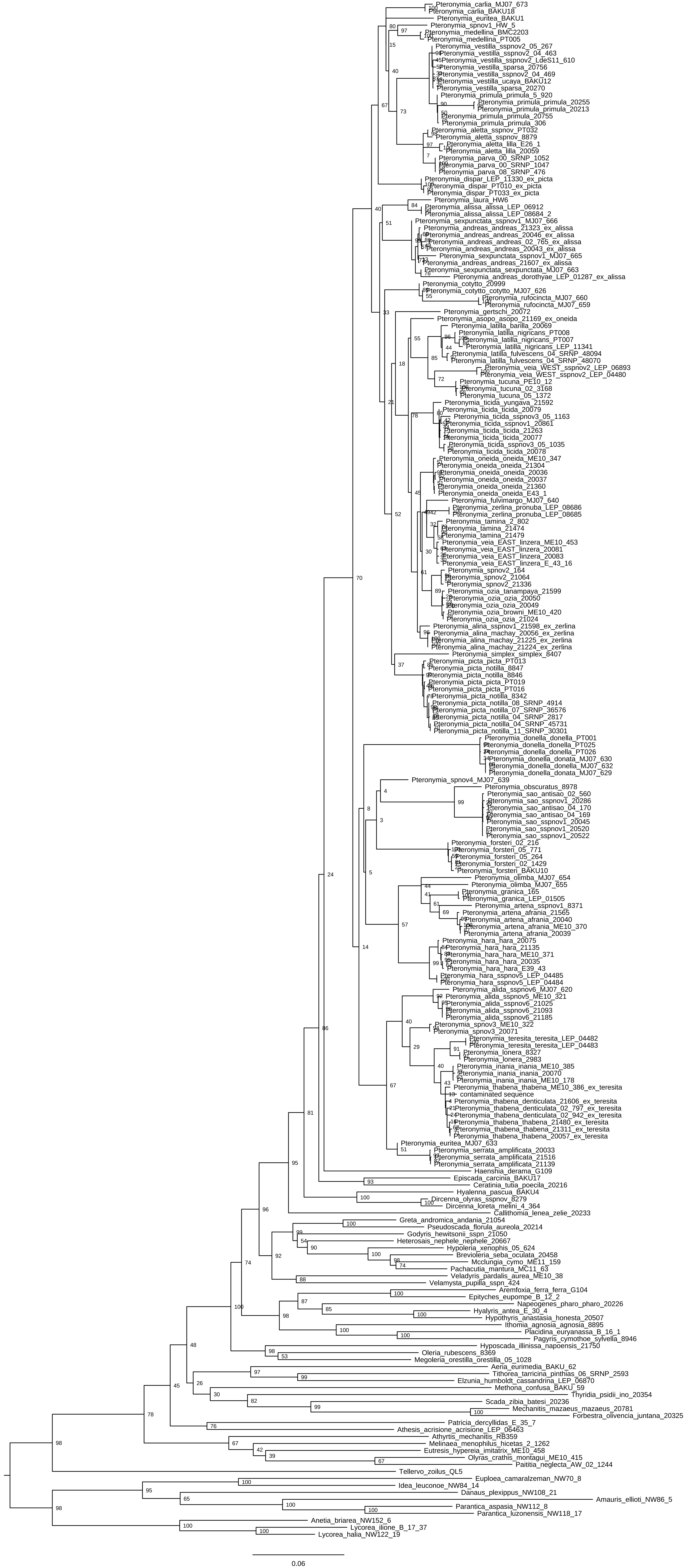
Pteronymia_carlia_MJ07_673
Pteronymia_carlia_BAKU18
Pteronymia_euritea_BAKU1
Pteronymia_spnov1_HW_5
Pteronymia_medellina_BMC2203
Pteronymia_medellina_PT005
Pteronymia_vestilla_sspnov2_05_267
Pteronymia_vestilla_sspnov2_04_463
Pteronymia_vestilla_sspnov2_LdeS11_610
Pteronymia_vestilla_sparsa_20756
Pteronymia_vestilla_sspnov2_04_469
Pteronymia_vestilla_ucaya_BAKU12
Pteronymia_vestilla_sparsa_20270
Pteronymia_primula_primula_5_920
Pteronymia_primula_primula_20255
Pteronymia_primula_primula_20213
Pteronymia_primula_primula_20755
Pteronymia_primula_primula_306
Pteronymia_aletta_sspnov_PT032
Pteronymia_aletta_sspnov_8879
Pteronymia_aletta_lilla_E26_1
Pteronymia_aletta_lilla_20059
Pteronymia_parva_00_SRNP_1052
Pteronymia_parva_00_SRNP_1047
Pteronymia_parva_08_SRNP_476
Pteronymia_dispar_LEP_11330_ex_picta
Pteronymia_dispar_PT010_ex_picta
Pteronymia_dispar_PT033_ex_picta
Pteronymia_laura_HW6
Pteronymia_alissa_alissa_LEP_06912
Pteronymia_alissa_alissa_LEP_08684_2
Pteronymia_sexpunctata_sspnov1_MJ07_666
Pteronymia_andreas_andreas_21323_ex_alissa
Pteronymia_andreas_andreas_20046_ex_alissa
Pteronymia_andreas_andreas_02_765_ex_alissa
Pteronymia_andreas_andreas_20043_ex_alissa
Pteronymia_sexpunctata_sspnov1_MJ07_665
Pteronymia_andreas_andreas_21607_ex_alissa
Pteronymia_sexpunctata_sexpunctata_MJ07_663
Pteronymia_andreas_dorothyae_LEP_01287_ex_alissa
Pteronymia_cotytto_20999
Pteronymia_cotytto_cotytto_MJ07_626
Pteronymia_rufocincta_MJ07_660
Pteronymia_rufocincta_MJ07_659
Pteronymia_gertschi_20072
Pteronymia_asopo_asopo_21169_ex_oneida
Pteronymia_latilla_barilla_20069
Pteronymia_latilla_nigricans_PT008
Pteronymia_latilla_nigricans_PT007
Pteronymia_latilla_nigricans_LEP_11341
Pteronymia_latilla_fulvescens_04_SRNP_48094
Pteronymia_latilla_fulvescens_04_SRNP_48070
Pteronymia_veia_WEST_sspnov2_LEP_06893
Pteronymia_veia_WEST_sspnov2_LEP_04480
Pteronymia_tucuna_PE10_12
Pteronymia_tucuna_02_3168
Pteronymia_tucuna_05_1372
Pteronymia_ticida_yungava_21592
Pteronymia_ticida_ticida_20079
Pteronymia_ticida_sspnov3_05_1163
Pteronymia_ticida_sspnov1_20861
Pteronymia_ticida_ticida_21263
Pteronymia_ticida_ticida_20077
Pteronymia_ticida_sspnov3_05_1035
Pteronymia_ticida_ticida_20078
Pteronymia_oneida_oneida_ME10_347
Pteronymia_oneida_oneida_21304
Pteronymia_oneida_oneida_20036
Pteronymia_oneida_oneida_20037
Pteronymia_oneida_oneida_21360
Pteronymia_oneida_oneida_E43_1
Pteronymia_fulvimargo_MJ07_640
Pteronymia_zerlina_pronuba_LEP_08686
Pteronymia_zerlina_pronuba_LEP_08685
Pteronymia_tamina_2_802
Pteronymia_tamina_21474
Pteronymia_tamina_21479
Pteronymia_veia_EAST_linzera_ME10_453
Pteronymia_veia_EAST_linzera_20081
Pteronymia_veia_EAST_linzera_20083
Pteronymia_veia_EAST_linzera_E_43_16
Pteronymia_spnov2_164
Pteronymia_spnov2_21064
Pteronymia_spnov2_21336
Pteronymia_ozia_tanampaya_21599
Pteronymia_ozia_ozia_20050
Pteronymia_ozia_ozia_20049
Pteronymia_ozia_browni_ME10_420
Pteronymia_ozia_ozia_21024
Pteronymia_alina_sspnov1_21598_ex_zerlina
Pteronymia_alina_machay_20056_ex_zerlina
Pteronymia_alina_machay_21225_ex_zerlina
Pteronymia_alina_machay_21224_ex_zerlina
Pteronymia_simplex_simplex_8407
Pteronymia_picta_picta_PT013
Pteronymia_picta_notilla_8847
Pteronymia_picta_notilla_8846
Pteronymia_picta_picta_PT019
Pteronymia_picta_picta_PT016
Pteronymia_picta_notilla_8342
Pteronymia_picta_notilla_08_SRNP_4914
Pteronymia_picta_notilla_07_SRNP_36576
Pteronymia_picta_notilla_04_SRNP_2817
Pteronymia_picta_notilla_04_SRNP_45731
Pteronymia_picta_notilla_11_SRNP_30301
Pteronymia_donella_donella_PT001
Pteronymia_donella_donella_PT025
Pteronymia_donella_donella_PT026
Pteronymia_donella_donata_MJ07_630
Pteronymia_donella_donella_MJ07_632
Pteronymia_donella_donella_MJ07_629
Pteronymia_spnov4_MJ07_639
Pteronymia_obscuratus_8978
Pteronymia_sao_antisao_02_560
Pteronymia_sao_sspnov1_20286
Pteronymia_sao_antisao_04_170
Pteronymia_sao_antisao_04_169
Pteronymia_sao_sspnov1_20045
Pteronymia_sao_sspnov1_20520
Pteronymia_sao_sspnov1_20522
Pteronymia_forsteri_02_216
Pteronymia_forsteri_05_771
Pteronymia_forsteri_05_264
Pteronymia_forsteri_02_1429
Pteronymia_forsteri_BAKU10
Pteronymia_olimba_MJ07_654
Pteronymia_olimba_MJ07_655
Pteronymia_granica_165
Pteronymia_granica_LEP_01505
Pteronymia_artena_sspnov1_8371
Pteronymia_artena_afrania_21565
Pteronymia_artena_afrania_20040
Pteronymia_artena_afrania_ME10_370
Pteronymia_artena_afrania_20039
Pteronymia_hara_hara_20075
Pteronymia_hara_hara_21135
Pteronymia_hara_hara_ME10_371
Pteronymia_hara_hara_20035
Pteronymia_hara_hara_E39_43
Pteronymia_hara_sspnov5_LEP_04485
Pteronymia_hara_sspnov5_LEP_04484
Pteronymia_alida_sspnov6_MJ07_620
Pteronymia_alida_sspnov5_ME10_321
Pteronymia_alida_sspnov6_21025
Pteronymia_alida_sspnov6_21093
Pteronymia_alida_sspnov6_21185
Pteronymia_spnov3_ME10_322
Pteronymia_spnov3_20071
Pteronymia_teresita_teresita_LEP_04482
Pteronymia_teresita_teresita_LEP_04483
Pteronymia_lonera_8327
Pteronymia_lonera_2983
Pteronymia_inania_inania_ME10_385
Pteronymia_inania_inania_20070
Pteronymia_inania_inania_ME10_178
Pteronymia_thabena_thabena_ME10_386_ex_teresita
contaminated sequence
Pteronymia_thabena_denticulata_21606_ex_teresita
Pteronymia_thabena_denticulata_02_797_ex_teresita
Pteronymia_thabena_denticulata_02_942_ex_teresita
Pteronymia_thabena_thabena_21480_ex_teresita
Pteronymia_thabena_thabena_21311_ex_teresita
Pteronymia_thabena_thabena_20057_ex_teresita
Pteronymia_euritea_MJ07_633
Pteronymia_serrata_amplificata_20033
Pteronymia_serrata_amplificata_21516
Pteronymia_serrata_amplificata_21139
Haenshia_derama_G109
Episcada_carcinia_BAKU17
Ceratinia_tutia_poecila_20216
Hyalenna_pascua_BAKU4
Dircenna_olyras_sspnov_8279
Dircenna_loreta_melini_4_364
Callithomia_lenea_zelie_20233
Greta_andromica_andania_21054
Pseudoscada_florula_aureola_20214
Godyris_hewitsoni_sspn_21050
Heterosais_nephele_nephele_20667
Hypoleria_xenophis_05_624
Brevioleria_seba_oculata_20458
Mcclungia_cymo_ME11_159
Pachacutia_mantura_MC11_63
Veladyris_pardalis_aurea_ME10_38
Velamysta_pupilla_sspn_424
Aremfoxia_ferra_ferra_G104
Epityches_eupompe_B_12_2
Napeogenes_pharo_pharo_20226
Hyalyris_antea_E_30_4
Hypothyris_anastasia_honesta_20507
Ithomia_agnosia_agnosia_8895
Placidina_euryanassa_B_16_1
Pagyris_cymothoe_sylvella_8946
Hyposcada_illinissa_napoensis_21750
Oleria_rubescens_8369
Megoleria_orestilla_orestilla_05_1028
Aeria_eurimedia_BAKU_62
Tithorea_tarricina_pinthias_06_SRNP_2593
Elzunia_humboldt_cassandrina_LEP_06870
Methona_confusa_BAKU_59
Thyridia_psidii_ino_20354
Scada_zibia_batesi_20236
Mechanitis_mazaeus_mazaeus_20781
Forbestra_olivencia_juntana_20325
Patricia_dercyllidas_E_35_7
Athesis_acrisione_acrisione_LEP_06463
Athyrtis_mechanitis_RB359
Melinaea_menophilus_hicetas_2_1262
Eutresis_hypereia_imitatrix_ME10_458
Olyras_crathis_montagui_ME10_415
Paititia_neglecta_AW_02_1244
Tellervo_zoilus_QL5
Euploea_camaralzeman_NW70_8
Idea_leuconoe_NW84_14
Danaus_plexippus_NW108_21
Amauris_ellioti_NW86_5
Parantica_aspasia_NW112_8
Parantica_luzonensis_NW118_17
Anetia_briarea_NW152_6
Lycorea_ilione_B_17_37
Lycorea_halia_NW122_19
0.06

Supplementary Figure S2: Phylogenetic tree of *Pteronymia* obtained with MrBayes (mrbayes.sourceforge.net/).

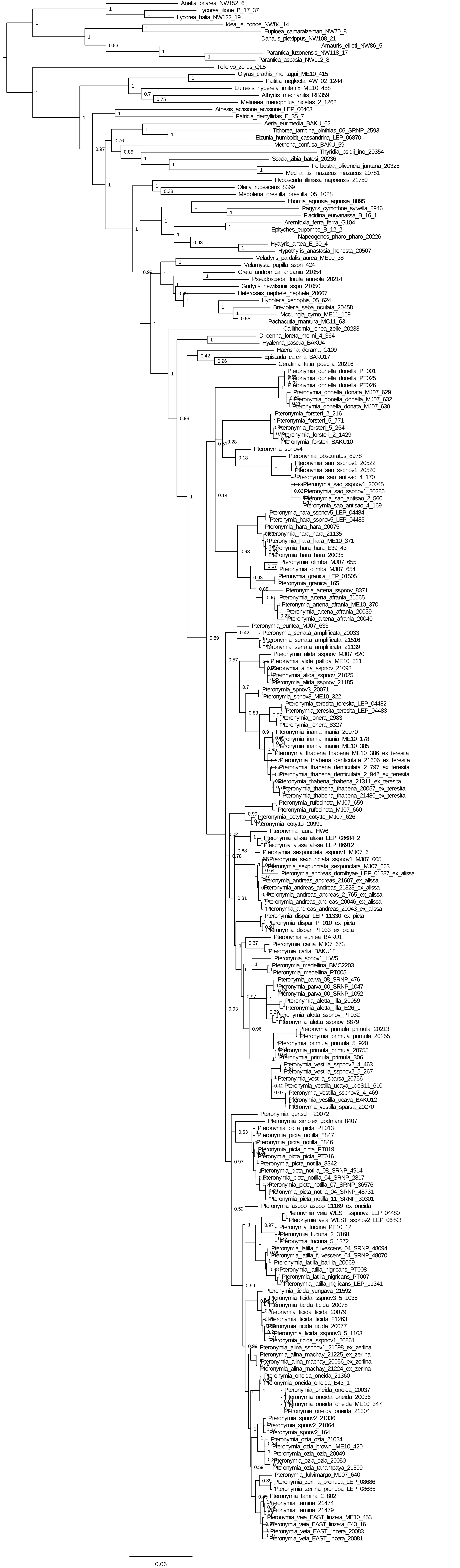

Anetia_briarea_NW152_6
Lycorea_ilione_B_17_37
Lycorea_halia_NW122_19
Idea_leuconoe_NW84_14
Euploea_camaralzeman_NW70_8
Danaus_plexippus_NW108_21
Amauris_ellioti_NW86_5
Parantica_luzonensis_NW118_17
Parantica_aspasia_NW112_8
Tellervo_zoilus_QL5
Olyras_crathis_montagui_ME10_415
Paititia_neglecta_AW_02_1244
Eutresis_hypereia_imitatrix_ME10_458
Athyrtis_mechanitis_RB359
Melinaea_menophilus_hicetas_2_1262
Athesis_acrisione_acrisione_LEP_06463
Patricia_dercyllidas_E_35_7
Aeria_eurimedia_BAKU_62
Tithorea_tarricina_pinthias_06_SRNP_2593
Elzunia_humboldt_cassandrina_LEP_06870
Methona_confusa_BAKU_59
Thyridia_psidii_ino_20354
Scada_zibia_batesi_20236
Forbestra_olivencia_juntana_20325
Mechanitis_mazaeus_mazaeus_20781
Hyposcada_illinissa_napoensis_21750
Oleria_rubescens_8369
Megoleria_orestilla_orestilla_05_1028
Ithomia_agnosia_agnosia_8895
Pagyris_cymothoe_sylvella_8946
Placidina_euryanassa_B_16_1
Aremfoxia_ferra_ferra_G104
Epityches_eupompe_B_12_2
Napeogenes_pharo_pharo_20226
Hyalyris_antea_E_30_4
Hypothyris_anastasia_honesta_20507
Veladyris_pardalis_aurea_ME10_38
Velamysta_pupilla_sspn_424
Greta_andromica_andania_21054
Pseudoscada_florula_aureola_20214
Godyris_hewitsonii_sspn_21050
Heterosais_nephele_nephele_20667
Hypoleria_xenophis_05_624
Brevioleria_seba_oculata_20458
Mcclungia_cymo_ME11_159
Pachacutia_mantura_MC11_63
Callithomia_lenea_zelie_20233
Dircenna_loreta_melini_4_364
Hyalenna_pascua_BAKU4
Haenshia_derama_G109
Episcada_carcinia_BAKU17
Ceratinia_tutia_poecila_20216
Pteronymia_donella_donella_PT001
Pteronymia_donella_donella_PT025
Pteronymia_donella_donella_PT026
Pteronymia_donella_donata_MJ07_629
Pteronymia_donella_donata_MJ07_632
Pteronymia_donella_donata_MJ07_630
Pteronymia_forsteri_2_216
Pteronymia_forsteri_5_771
Pteronymia_forsteri_5_264
Pteronymia_forsteri_2_1429
Pteronymia_forsteri_BAKU10
Pteronymia_spnov4
Pteronymia_obscuratus_8978
Pteronymia_sao_sspnov1_20522
Pteronymia_sao_sspnov1_20520
Pteronymia_sao_antisao_4_170
Pteronymia_sao_sspnov1_20045
Pteronymia_sao_sspnov1_20286
Pteronymia_sao_antisao_2_560
Pteronymia_sao_antisao_4_169
Pteronymia_hara_sspnov5_LEP_04484
Pteronymia_hara_sspnov5_LEP_04485
Pteronymia_hara_hara_20075
Pteronymia_hara_hara_21135
Pteronymia_hara_hara_ME10_371
Pteronymia_hara_hara_E39_43
Pteronymia_hara_hara_20035
Pteronymia_olimba_MJ07_655
Pteronymia_olimba_MJ07_654
Pteronymia_granica_LEP_01505
Pteronymia_granica_165
Pteronymia_artena_sspnov_8371
Pteronymia_artena_afrania_21565
Pteronymia_artena_afrania_ME10_370
Pteronymia_artena_afrania_20039
Pteronymia_artena_afrania_20040
Pteronymia_euritea_MJ07_633
Pteronymia_serrata_amplificata_20033
Pteronymia_serrata_amplificata_21516
Pteronymia_serrata_amplificata_21139
Pteronymia_alida_sspnov_MJ07_620
Pteronymia_alida_pallida_ME10_321
Pteronymia_alida_sspnov_21093
Pteronymia_alida_sspnov_21025
Pteronymia_alida_sspnov_21185
Pteronymia_spnov3_20071
Pteronymia_spnov3_ME10_322
Pteronymia_teresita_teresita_LEP_04482
Pteronymia_teresita_teresita_LEP_04483
Pteronymia_lonera_2983
Pteronymia_lonera_8327
Pteronymia_inania_inania_20070
Pteronymia_inania_inania_ME10_178
Pteronymia_inania_inania_ME10_385
Pteronymia_thabena_thabena_ME10_386_ex_teresita
Pteronymia_thabena_denticulata_21606_ex_teresita
Pteronymia_thabena_denticulata_2_797_ex_teresita
Pteronymia_thabena_denticulata_2_942_ex_teresita
Pteronymia_thabena_thabena_21311_ex_teresita
Pteronymia_thabena_thabena_20057_ex_teresita
Pteronymia_thabena_thabena_21480_ex_teresita
Pteronymia_rufocincta_MJ07_659
Pteronymia_rufocincta_MJ07_660
Pteronymia_cotytto_cotytto_MJ07_626
Pteronymia_cotytto_20999
Pteronymia_laura_HW6
Pteronymia_alissa_alissa_LEP_08684_2
Pteronymia_alissa_alissa_LEP_06912
Pteronymia_sexpunctata_sspnov1_MJ07_6
Pteronymia_sexpunctata_sspnov1_MJ07_665
Pteronymia_sexpunctata_sexpunctata_MJ07_663
Pteronymia_andreas_dorothyae_LEP_01287_ex_alissa
Pteronymia_andreas_andreas_21607_ex_alissa
Pteronymia_andreas_andreas_21323_ex_alissa
Pteronymia_andreas_andreas_2_765_ex_alissa
Pteronymia_andreas_andreas_20046_ex_alissa
Pteronymia_andreas_andreas_20043_ex_alissa
Pteronymia_dispar_LEP_11330_ex_picta
Pteronymia_dispar_PT010_ex_picta
Pteronymia_dispar_PT033_ex_picta
Pteronymia_euritea_BAKU1
Pteronymia_carlia_MJ07_673
Pteronymia_carlia_BAKU18
Pteronymia_spnov1_HW5
Pteronymia_medellina_BMC2203
Pteronymia_medellina_PT005
Pteronymia_parva_08_SRNP_476
Pteronymia_parva_00_SRNP_1047
Pteronymia_parva_00_SRNP_1052
Pteronymia_aletta_lilla_20059
Pteronymia_aletta_lilla_E26_1
Pteronymia_aletta_sspnov_PT032
Pteronymia_aletta_sspnov_8879
Pteronymia_primula_primula_20213
Pteronymia_primula_primula_20255
Pteronymia_primula_primula_5_920
Pteronymia_primula_primula_20755
Pteronymia_primula_primula_306
Pteronymia_vestilla_sspnov2_4_463
Pteronymia_vestilla_sspnov2_5_267
Pteronymia_vestilla_sparsa_20756
Pteronymia_vestilla_ucayal_LdeS11_610
Pteronymia_vestilla_sspnov2_4_469
Pteronymia_vestilla_ucayal_BAKU12
Pteronymia_vestilla_sparsa_20270
Pteronymia_gertschi_20072
Pteronymia_simplex_godmani_8407
Pteronymia_picta_picta_PT013
Pteronymia_picta_notilla_8847
Pteronymia_picta_notilla_8846
Pteronymia_picta_picta_PT019
Pteronymia_picta_picta_PT016
Pteronymia_picta_notilla_8342
Pteronymia_picta_notilla_08_SRNP_4914
Pteronymia_picta_notilla_04_SRNP_2817
Pteronymia_picta_notilla_07_SRNP_36576
Pteronymia_picta_notilla_04_SRNP_45731
Pteronymia_picta_notilla_11_SRNP_30301
Pteronymia_asopo_asopo_21169_ex_oneida
Pteronymia_veia_WEST_sspnov2_LEP_04480
Pteronymia_veia_WEST_sspnov2_LEP_06893
Pteronymia_tucuna_PE10_12
Pteronymia_tucuna_2_3168
Pteronymia_tucuna_5_1372
Pteronymia_latilla_fulvescens_04_SRNP_48094
Pteronymia_latilla_fulvescens_04_SRNP_48070
Pteronymia_latilla_barilla_20069
Pteronymia_latilla_nigricans_PT008
Pteronymia_latilla_nigricans_PT007
Pteronymia_latilla_nigricans_LEP_11341
Pteronymia_ticida_yungava_21592
Pteronymia_ticida_sspnov3_5_1035
Pteronymia_ticida_ticida_20078
Pteronymia_ticida_ticida_20079
Pteronymia_ticida_ticida_21263
Pteronymia_ticida_ticida_20077
Pteronymia_ticida_sspnov3_5_1163
Pteronymia_ticida_sspnov1_20861
Pteronymia_alina_sspnov1_21598_ex_zerlina
Pteronymia_alina_machay_21225_ex_zerlina
Pteronymia_alina_machay_20056_ex_zerlina
Pteronymia_alina_machay_21224_ex_zerlina
Pteronymia_oneida_oneida_21360
Pteronymia_oneida_oneida_E43_1
Pteronymia_oneida_oneida_20037
Pteronymia_oneida_oneida_20036
Pteronymia_oneida_oneida_ME10_347
Pteronymia_oneida_oneida_21304
Pteronymia_spnov2_21336
Pteronymia_spnov2_21064
Pteronymia_spnov2_164
Pteronymia_ozia_ozia_21024
Pteronymia_ozia_browni_ME10_420
Pteronymia_ozia_ozia_20049
Pteronymia_ozia_ozia_20050
Pteronymia_ozia_tanampaya_21599
Pteronymia_fulvimargo_MJ07_640
Pteronymia_zerlina_pronuba_LEP_08686
Pteronymia_zerlina_pronuba_LEP_08685
Pteronymia_tamina_2_802
Pteronymia_tamina_21474
Pteronymia_tamina_21479
Pteronymia_veia_EAST_linzera_ME10_453
Pteronymia_veia_EAST_linzera_E43_16
Pteronymia_veia_EAST_linzera_20083
Pteronymia_veia_EAST_linzera_20081
0.06

Supplementary figure S3. **Maximum parsimony phylogeny based on morphological characters using TNT (www.zmuc.dk/public/phylogeny/tnt/).**
Majority rule consensus with bootstrap and Bremer supports.

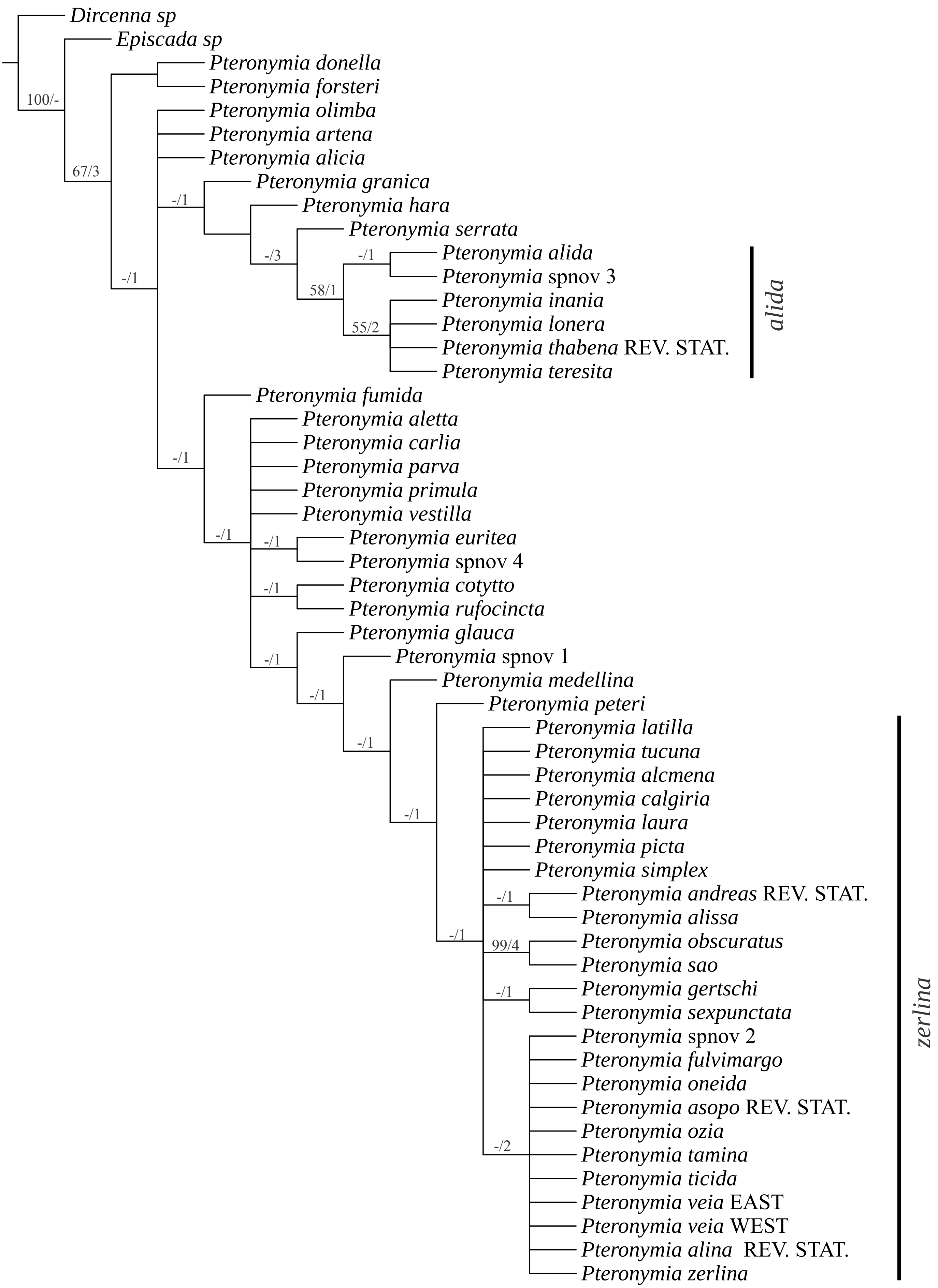

Supplementary Figure S4. Recalibrated phylogeny of Solanaceae generated with BEAST 1.8.2 (beast.bio.ed.ac.uk/).

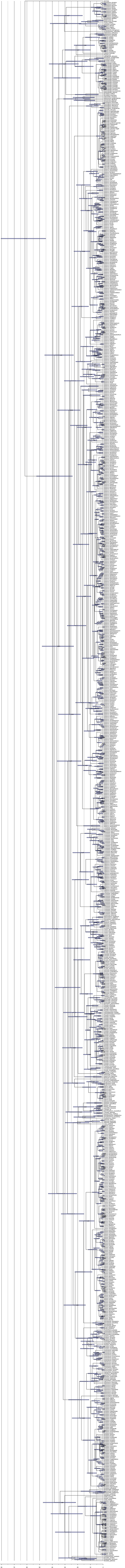

Supplementary Figure S5. Distribution maps and photos of described *Pteronymia* species. Maps were generated with R (https://cran.r-project.org/); photos were taken by Keith Willmott. The figure was edited using Adobe Acrobat XI Pro (https://acrobat.adobe.com).

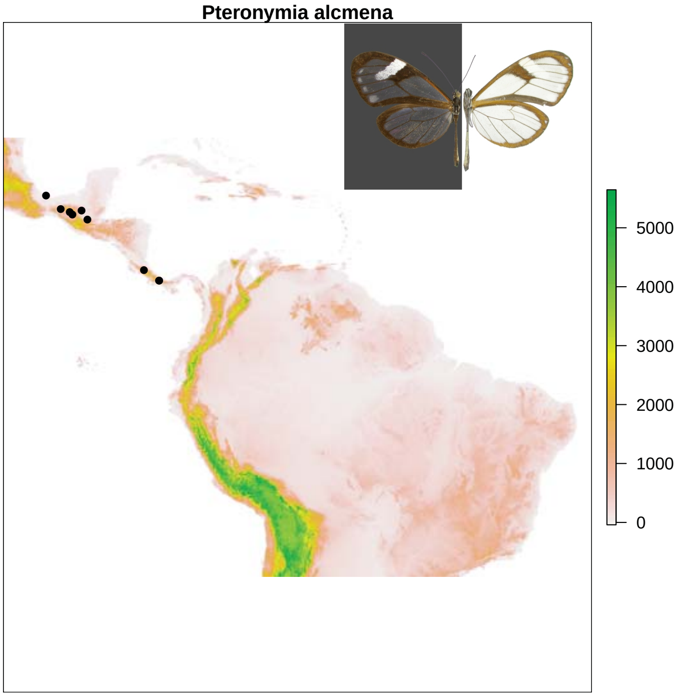
Pteronymia alcmena
5000
4000
3000
2000
1000
0

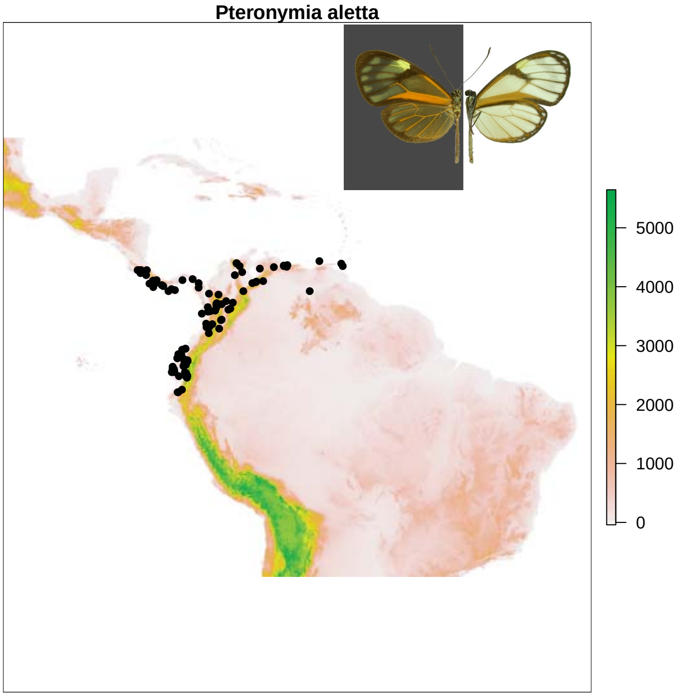
Pteronymia aletta
5000
4000
3000
2000
1000
0

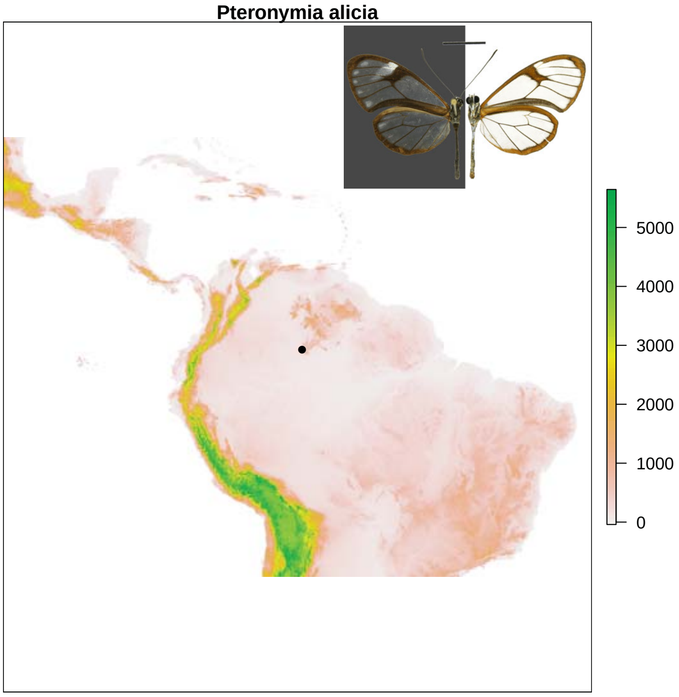
Pteronymia alicia
5000
4000
3000
2000
1000
0

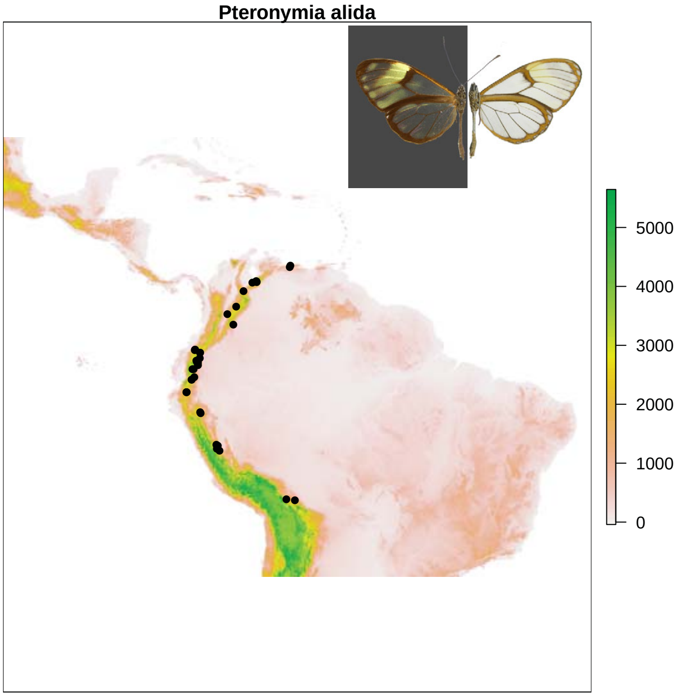
Pteronymia alida
5000
4000
3000
2000
1000
0

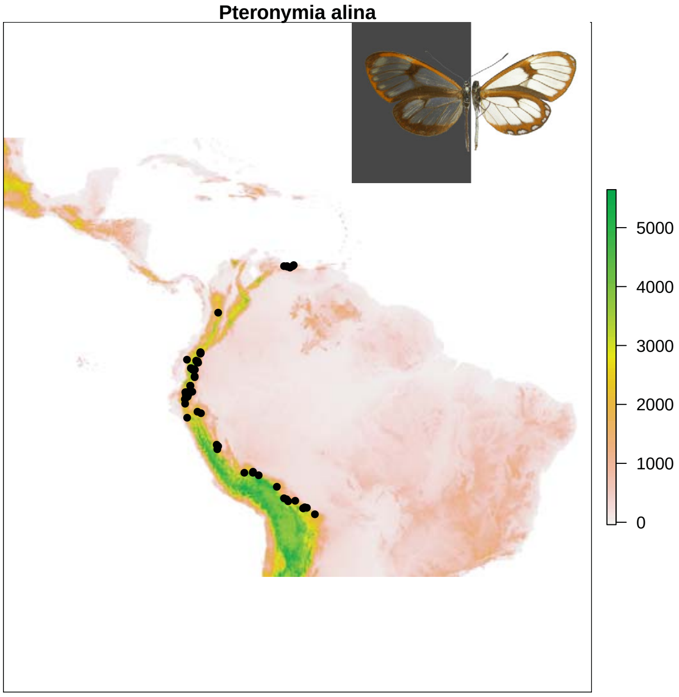
Pteronymia alina
5000
4000
3000
2000
1000
0

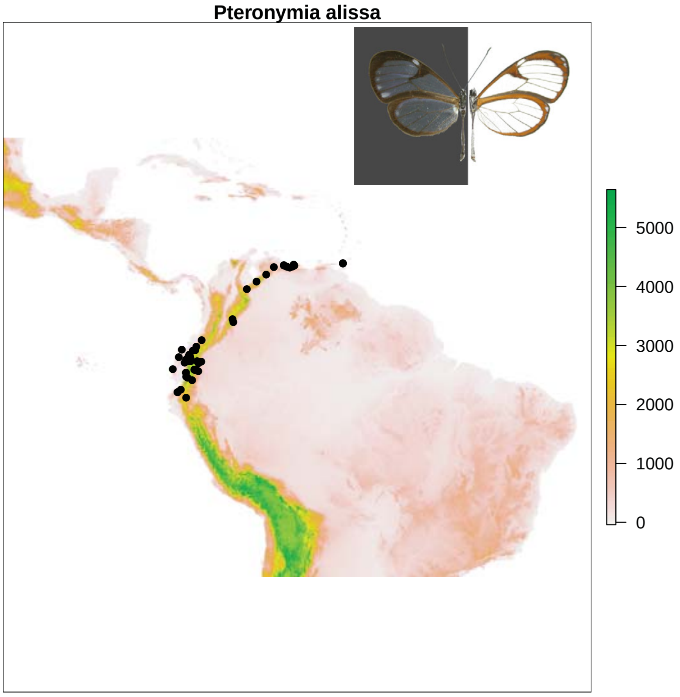
Pteronymia alissa
5000
4000
3000
2000
1000
0

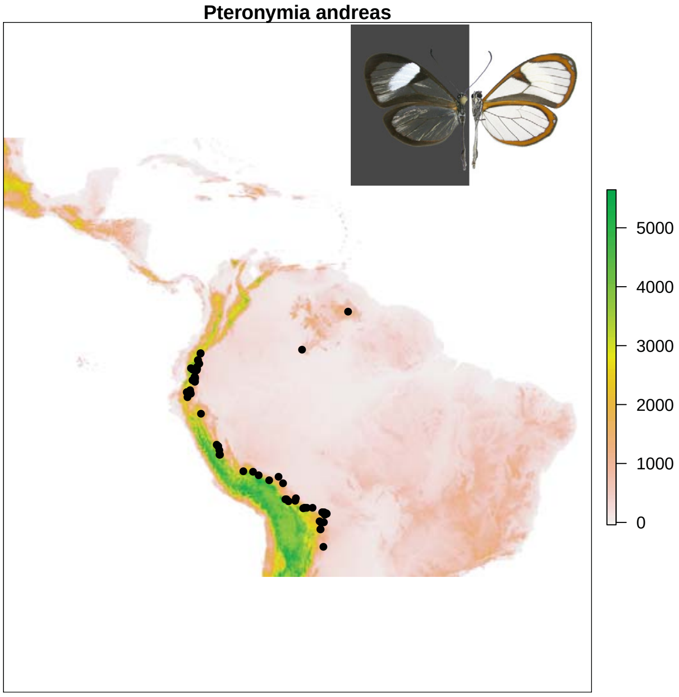
Pteronymia andreas
5000
4000
3000
2000
1000
0

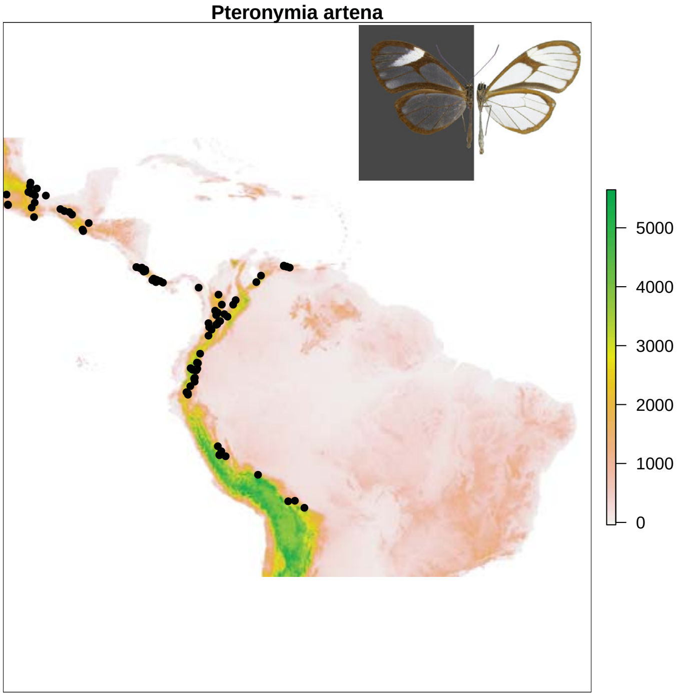
Pteronymia artena
5000
4000
3000
2000
1000
0

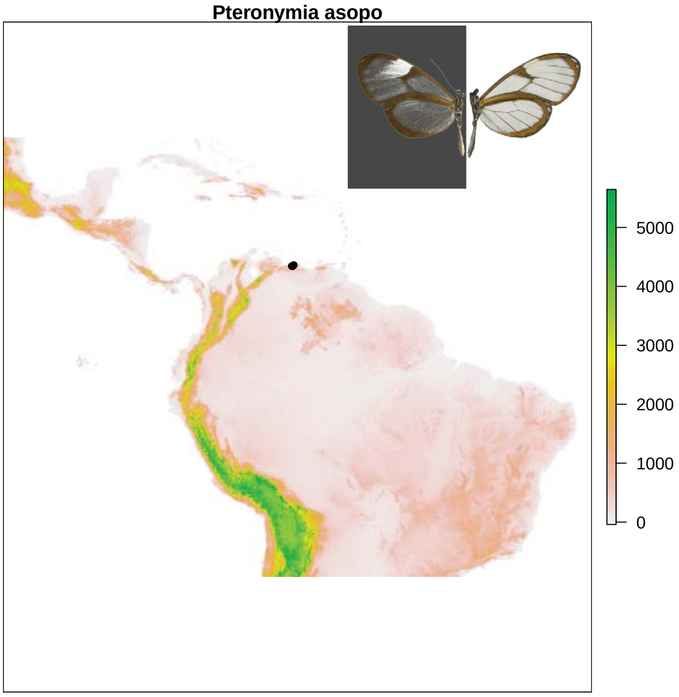
Pteronymia asopo
5000
4000
3000
2000
1000
0

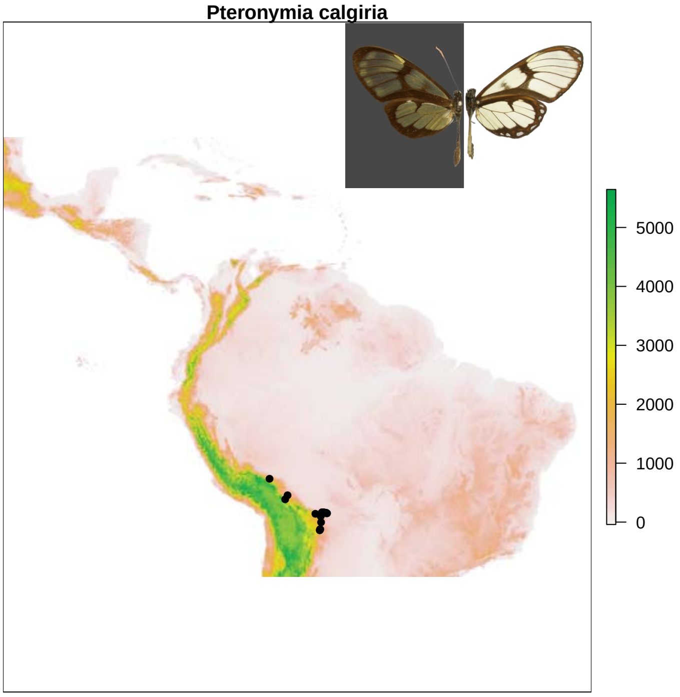
Pteronymia calgiria
5000
4000
3000
2000
1000
0

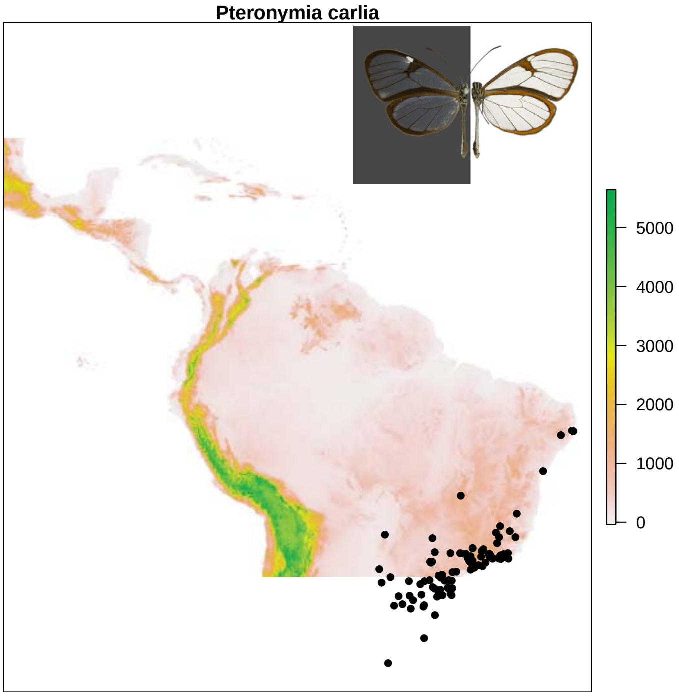
Pteronymia carlia
5000
4000
3000
2000
1000
0

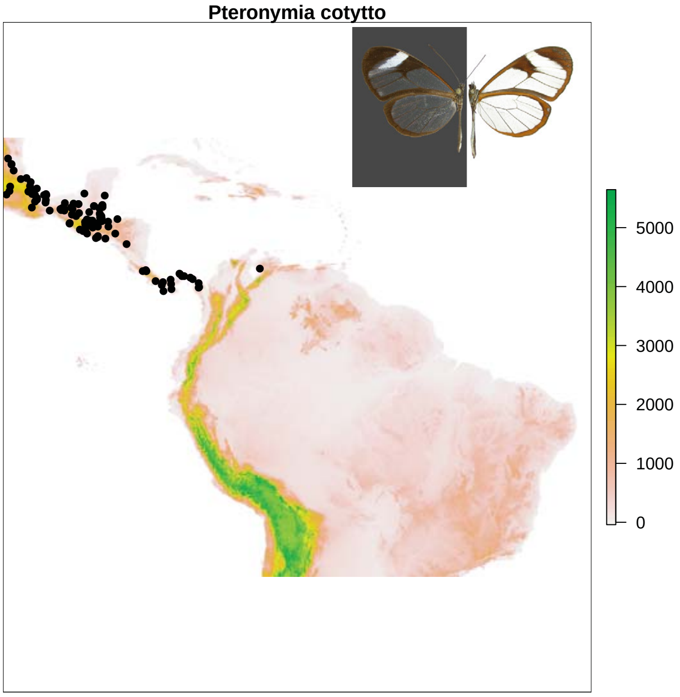
Pteronymia cotytto
5000
4000
3000
2000
1000
0

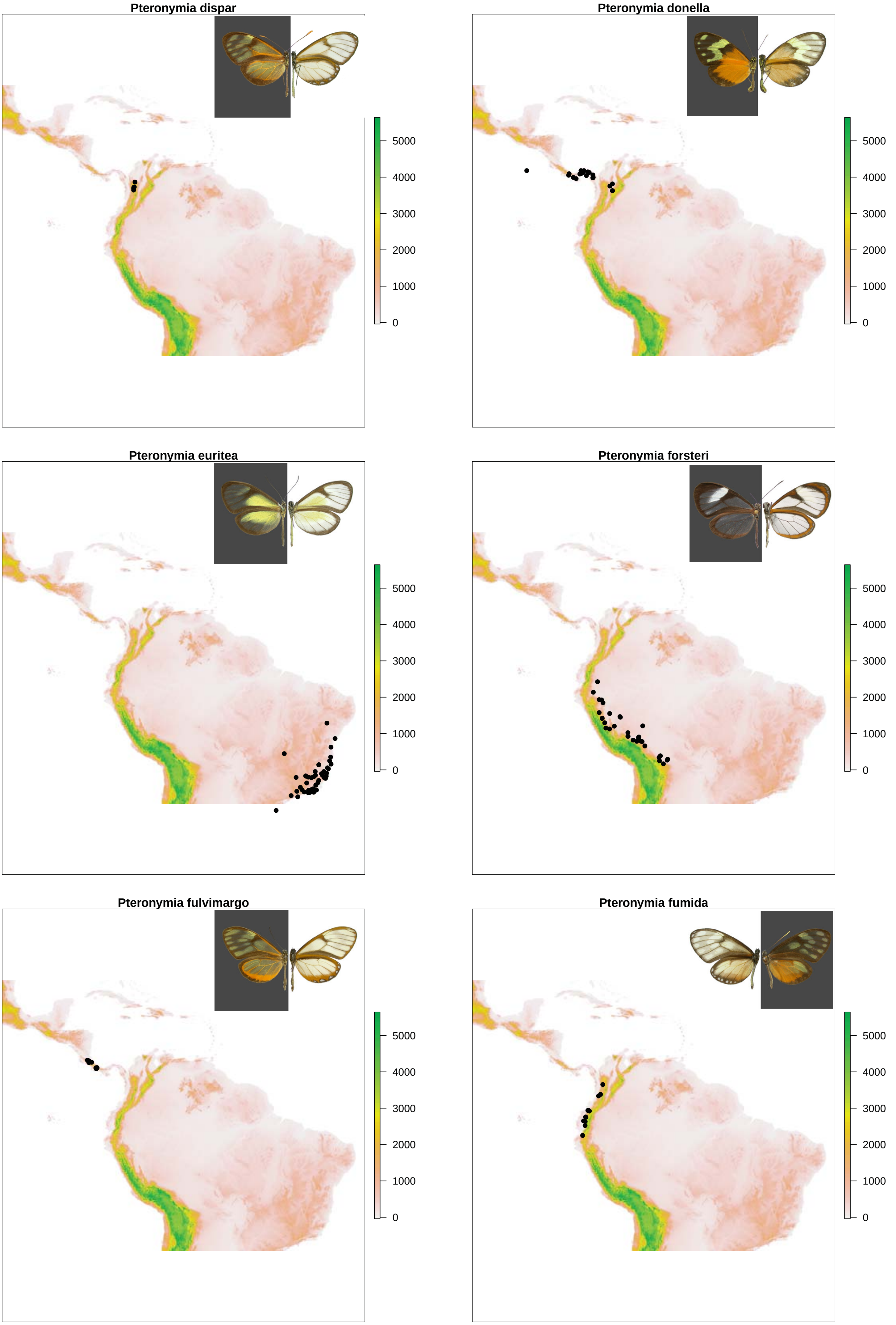
Pteronymia dispar
Pteronymia donella
Pteronymia euritea
Pteronymia forsteri
Pteronymia fulvimargo
Pteronymia fumida
5000
4000
3000
2000
1000
0

Pteronymia gertschi

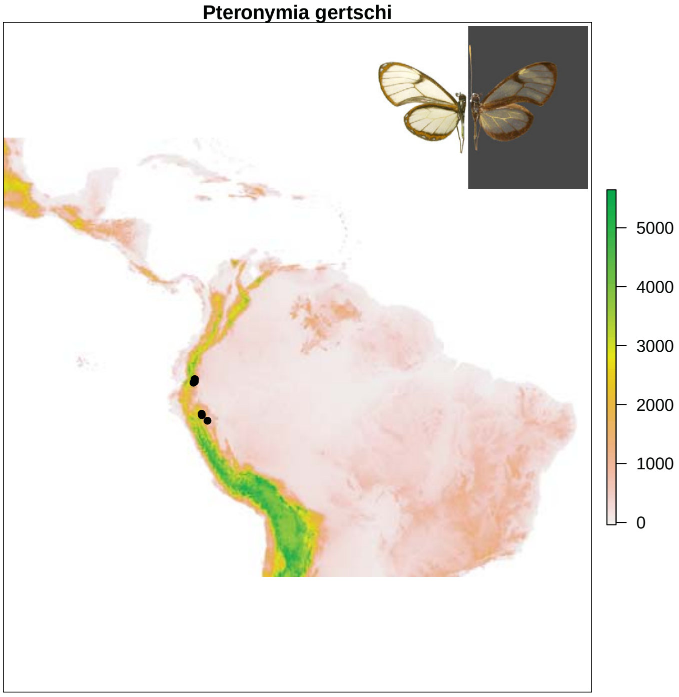

Pteronymia glauca

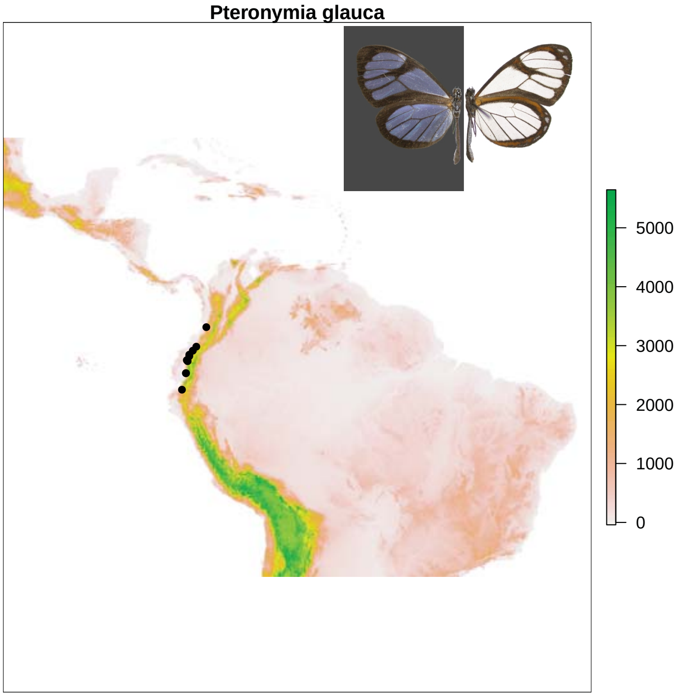

Pteronymia granica

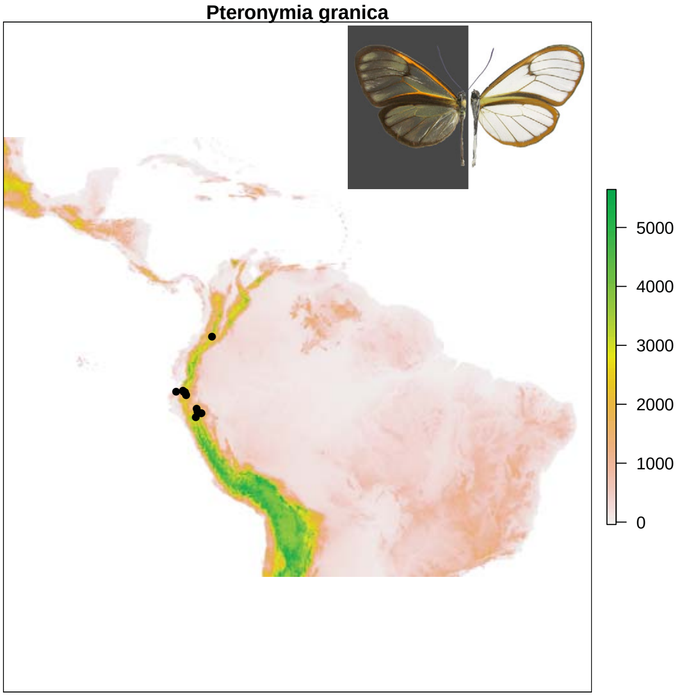

Pteronymia hara

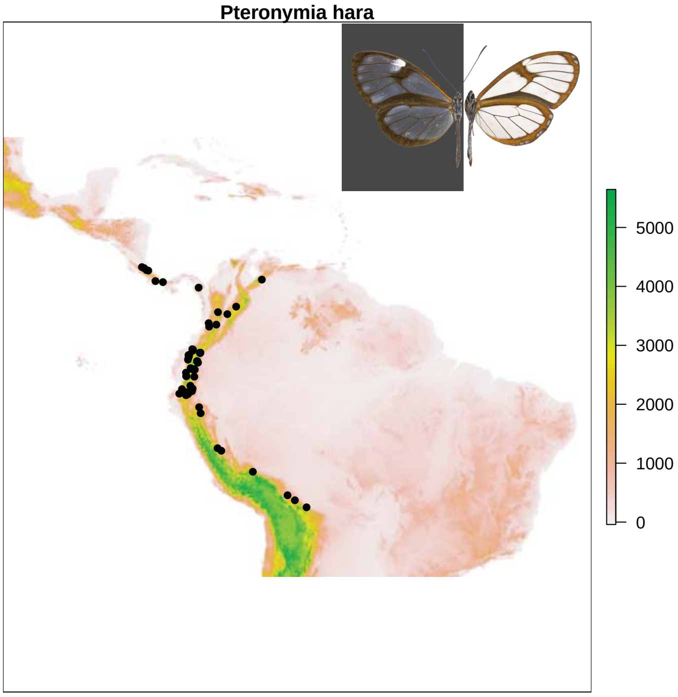

Pteronymia inania

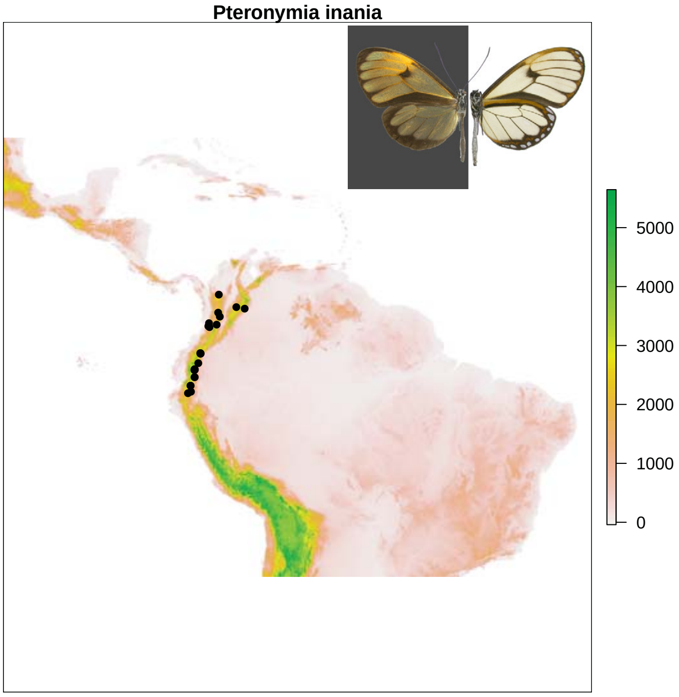

Pteronymia latilla

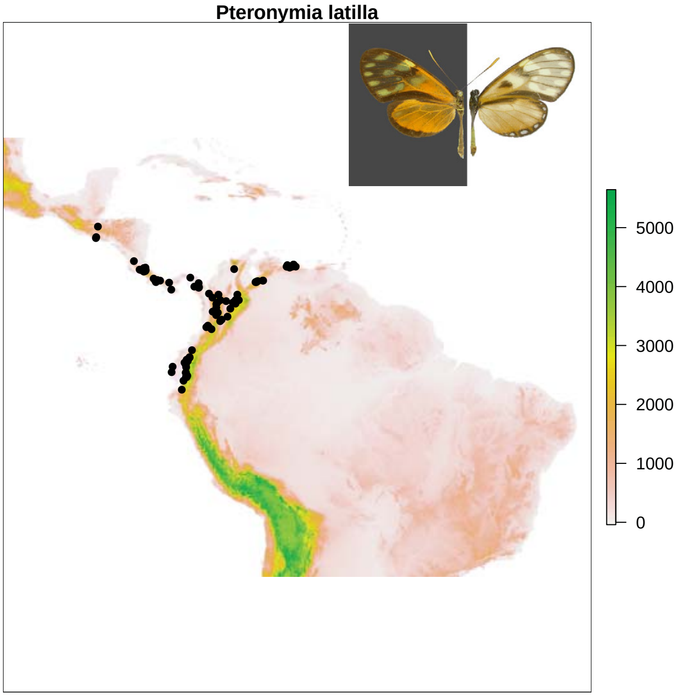

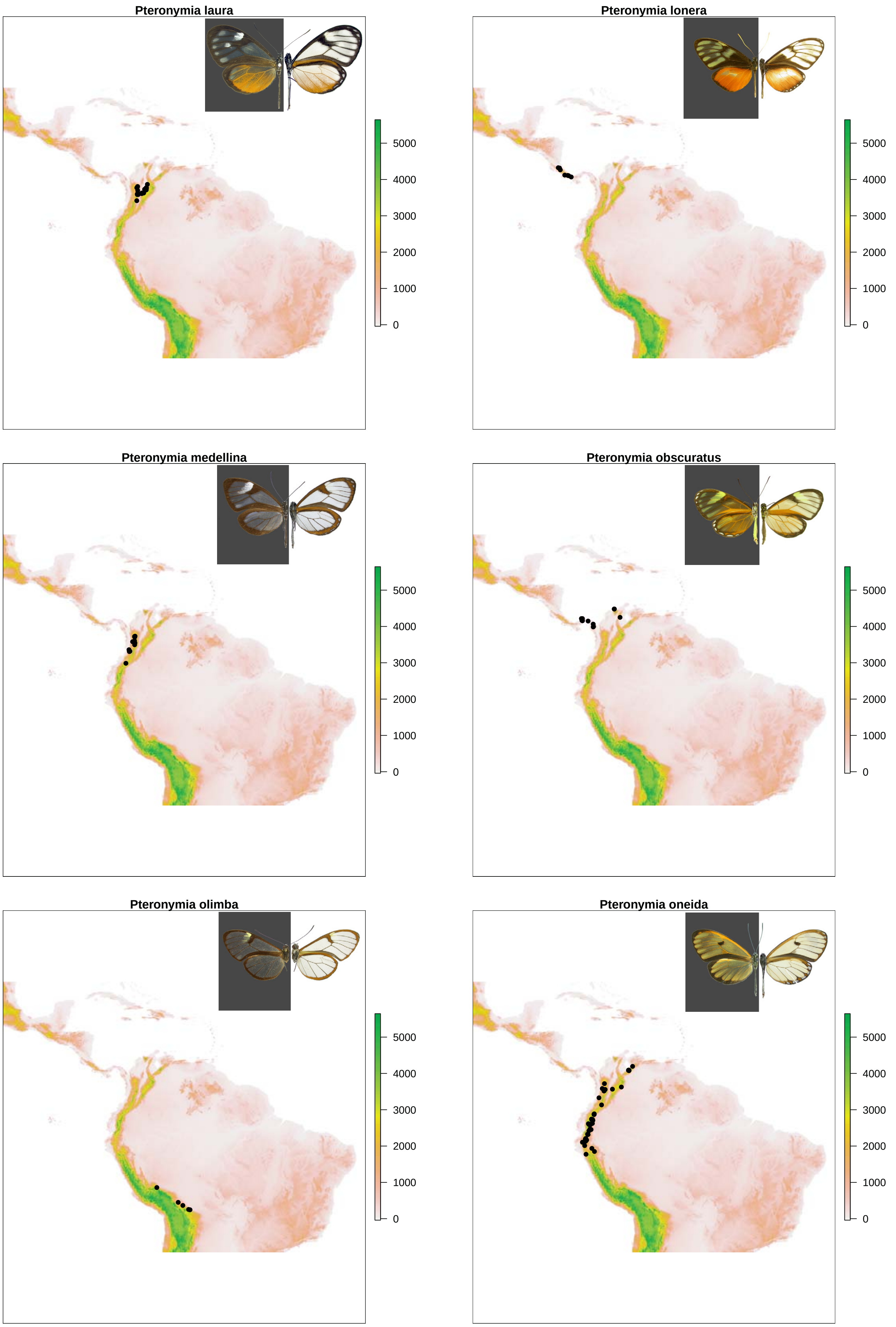
Pteronymia laura
Pteronymia lonera
Pteronymia medellina
Pteronymia obscuratus
Pteronymia olimba
Pteronymia oneida
5000
4000
3000
2000
1000
0

Pteronymia ozia

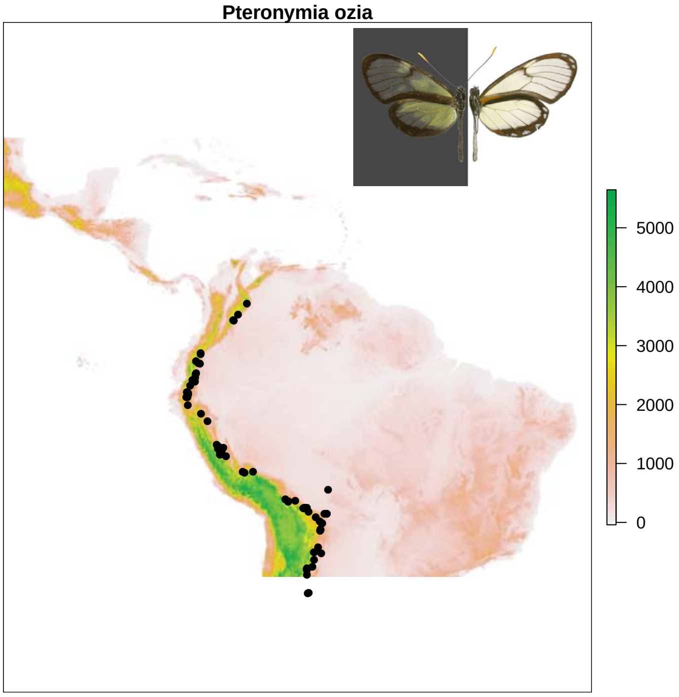

Pteronymia parva

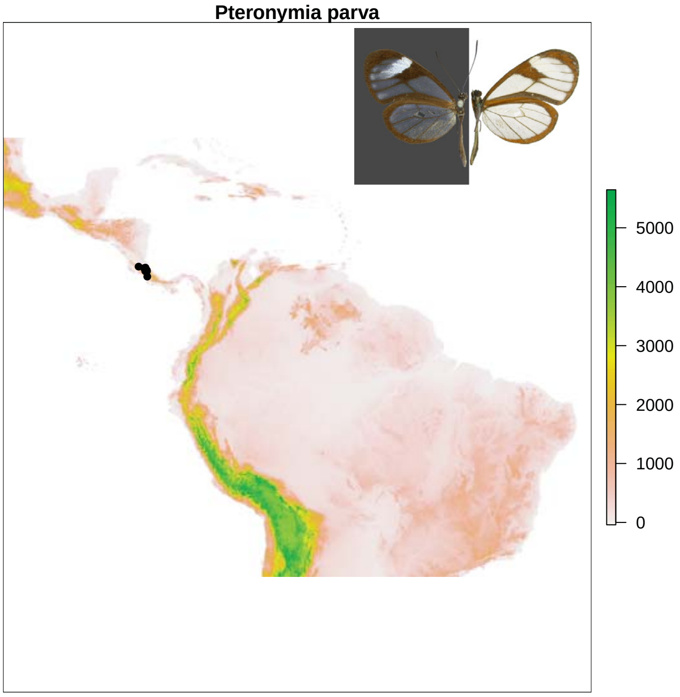

Pteronymia peteri

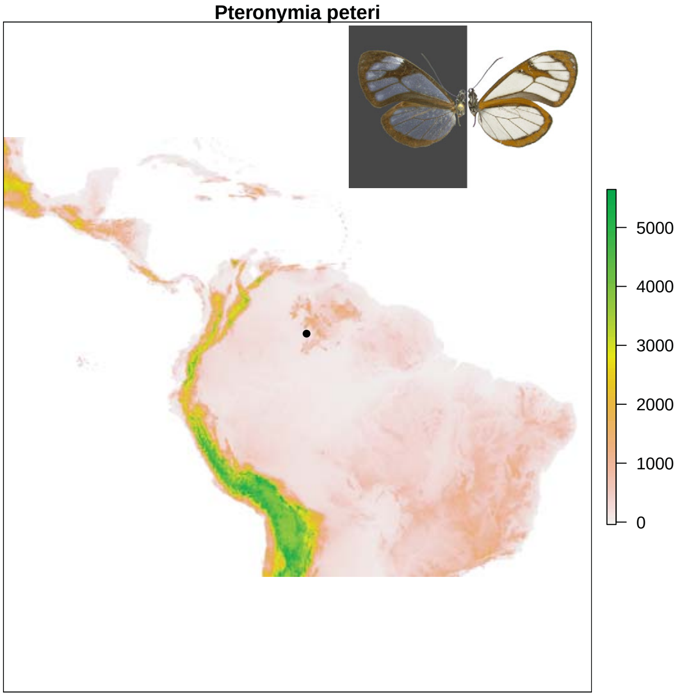

Pteronymia picta

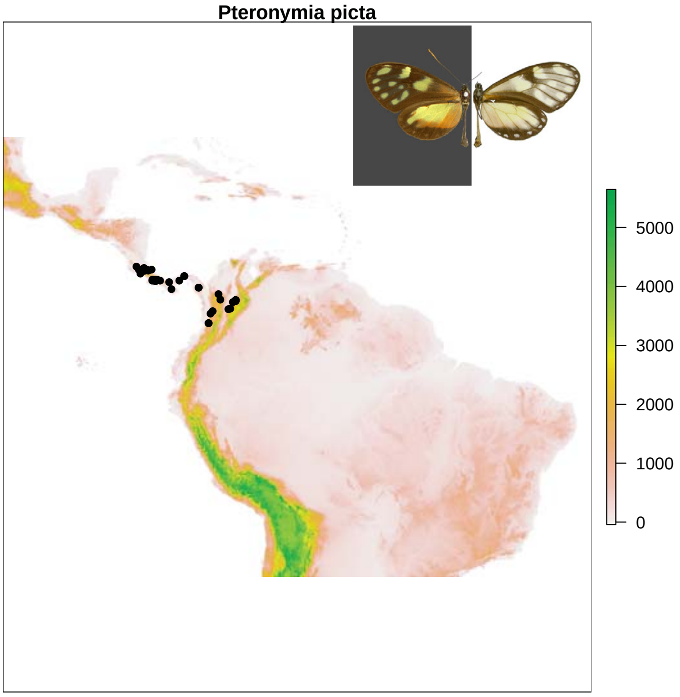

Pteronymia primula

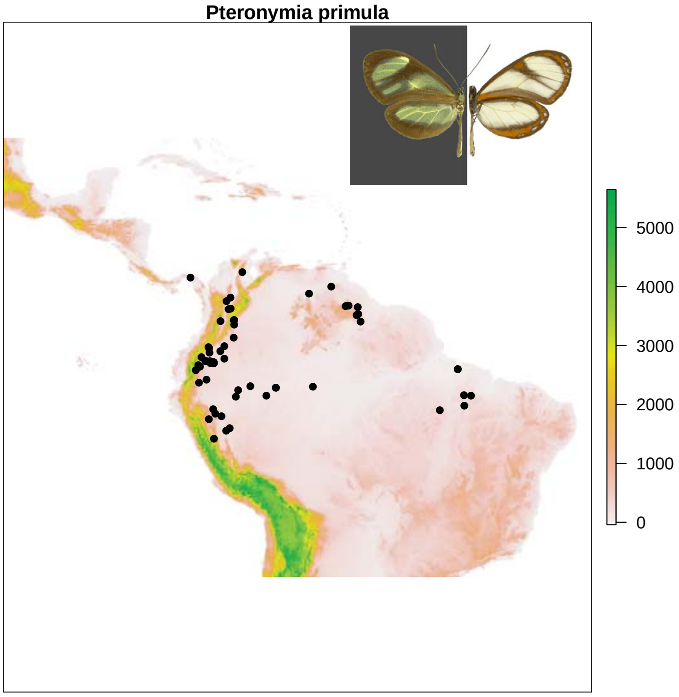

Pteronymia rufocincta

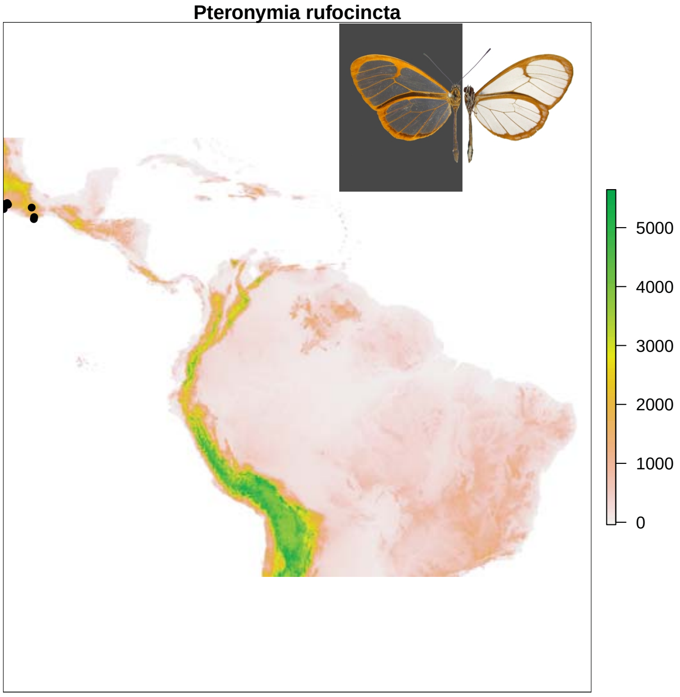

Pteronymia sao

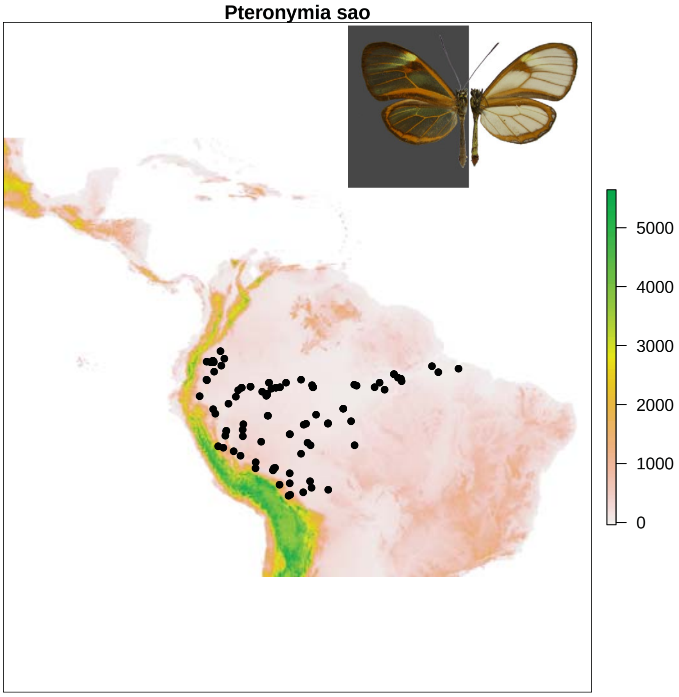

Pteronymia serrata

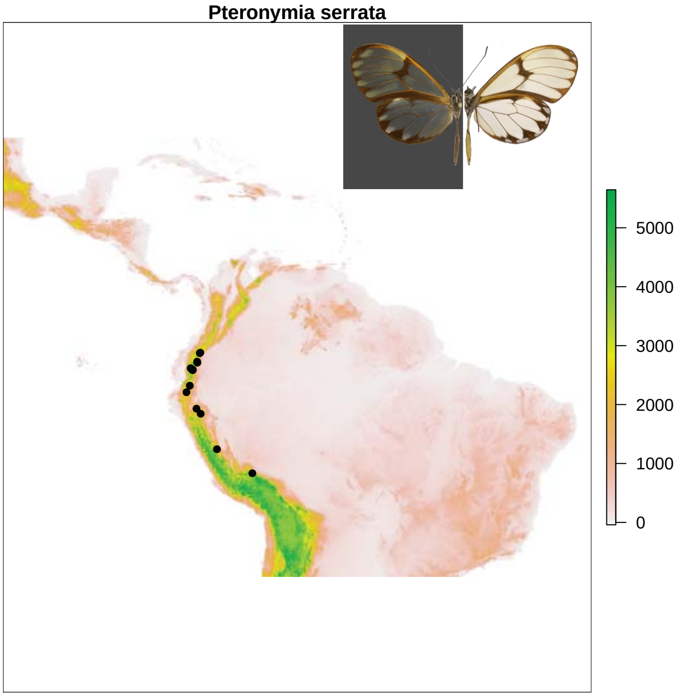

Pteronymia sexpunctata

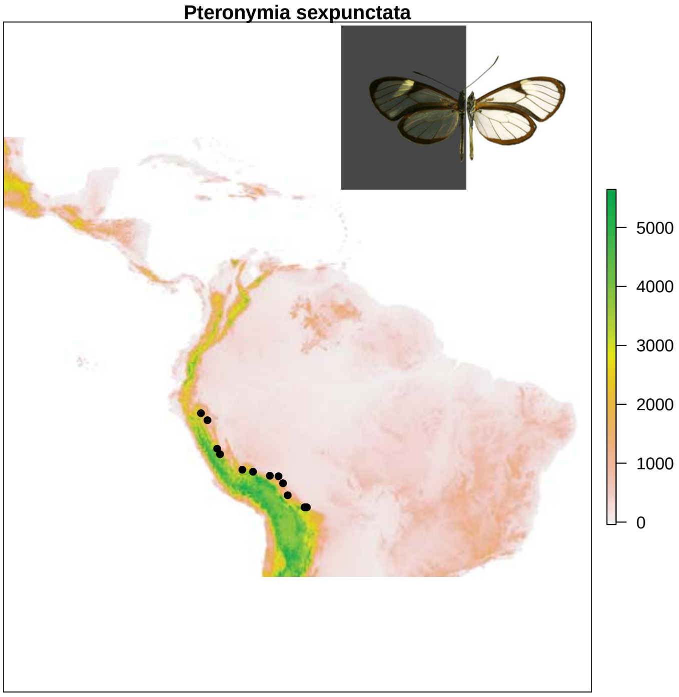

Pteronymia simplex

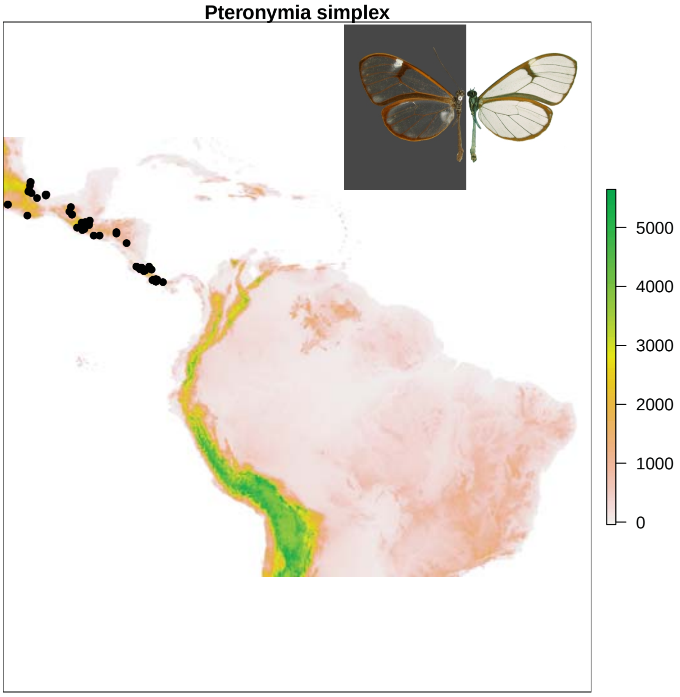

Pteronymia tamina

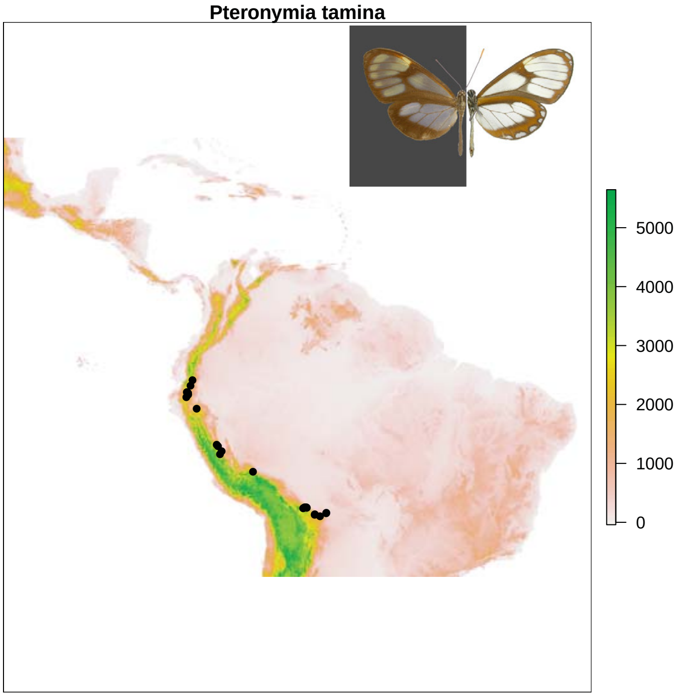

Pteronymia teresita

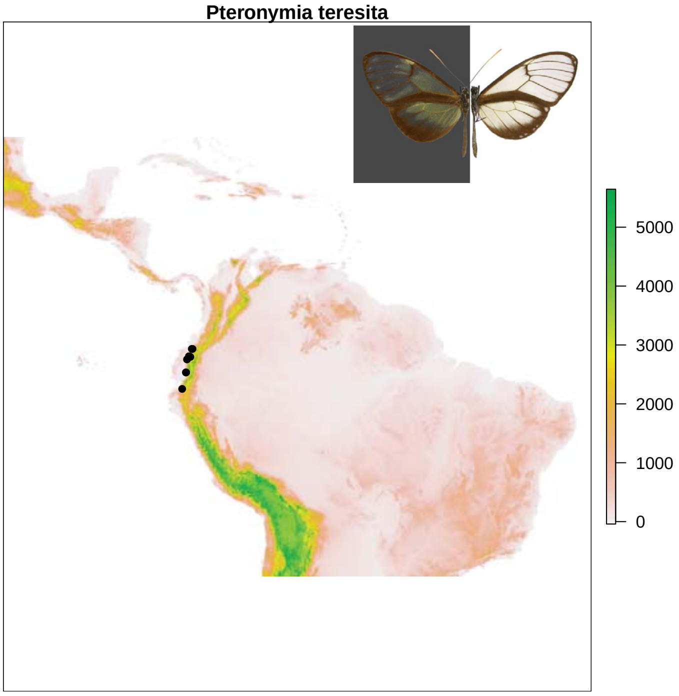

Pteronymia thabena

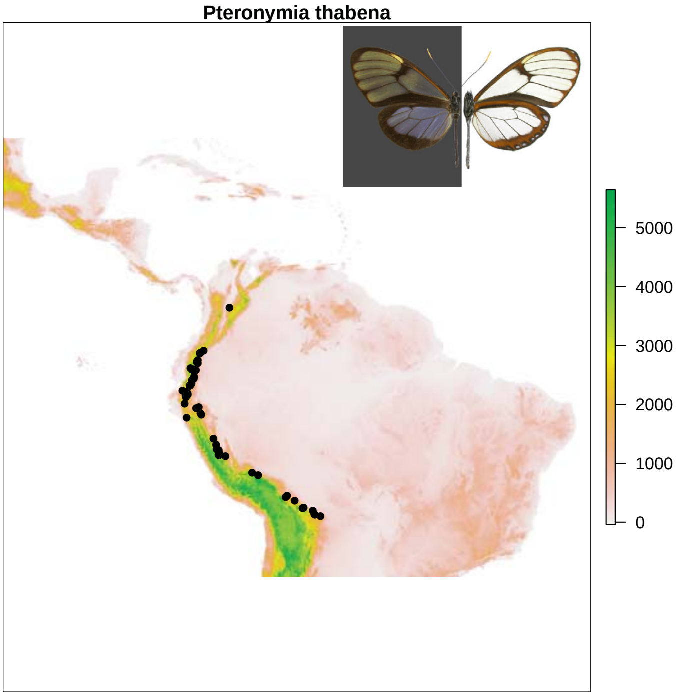

Pteronymia ticida

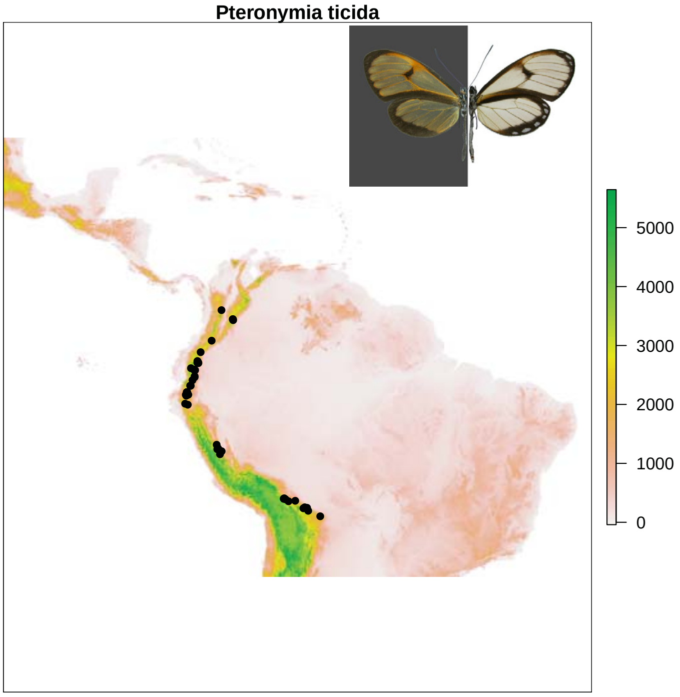

Pteronymia tucuna

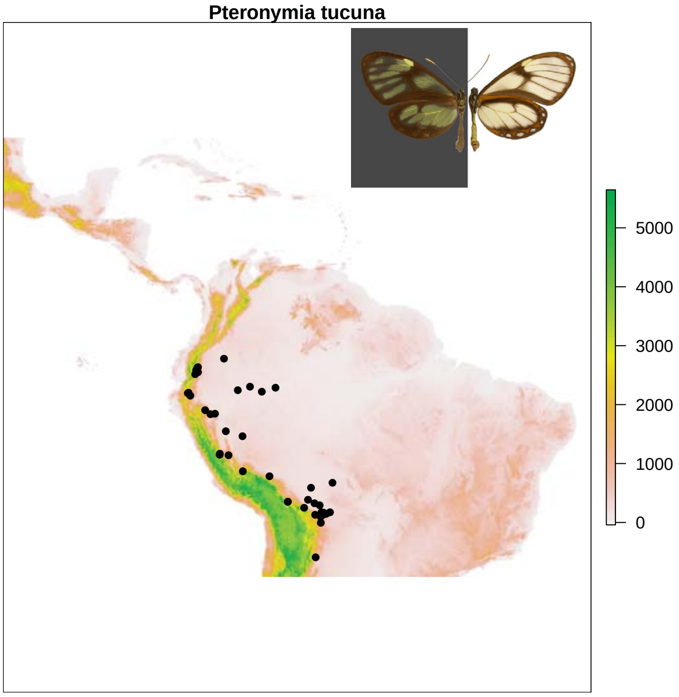

Pteronymia veia EAST

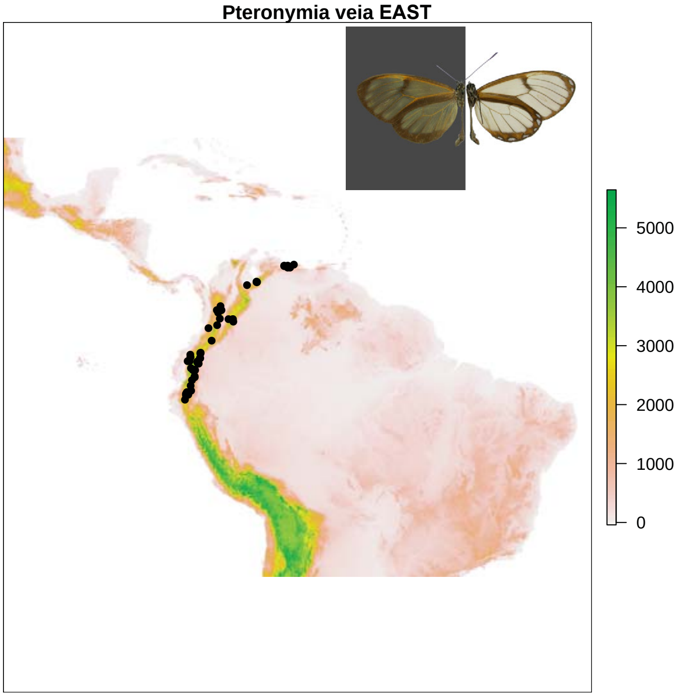

Pteronymia vestilla

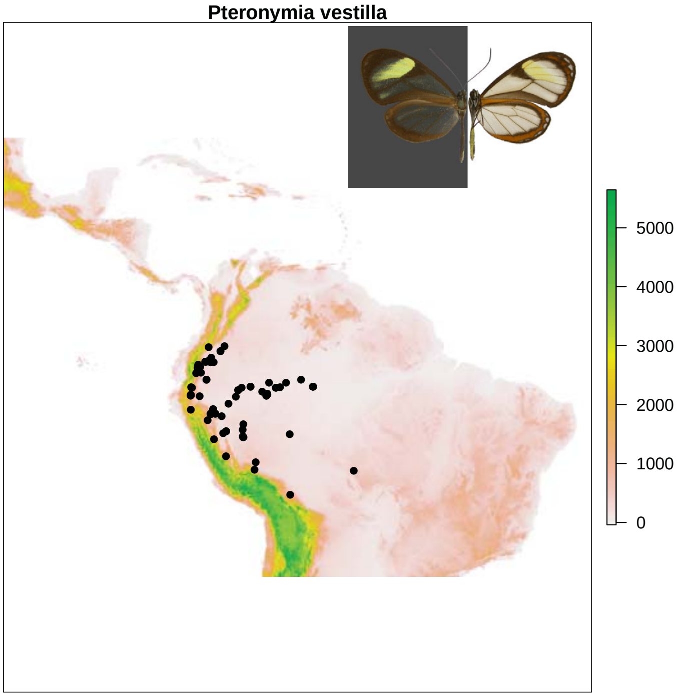

Pteronymia zerlina

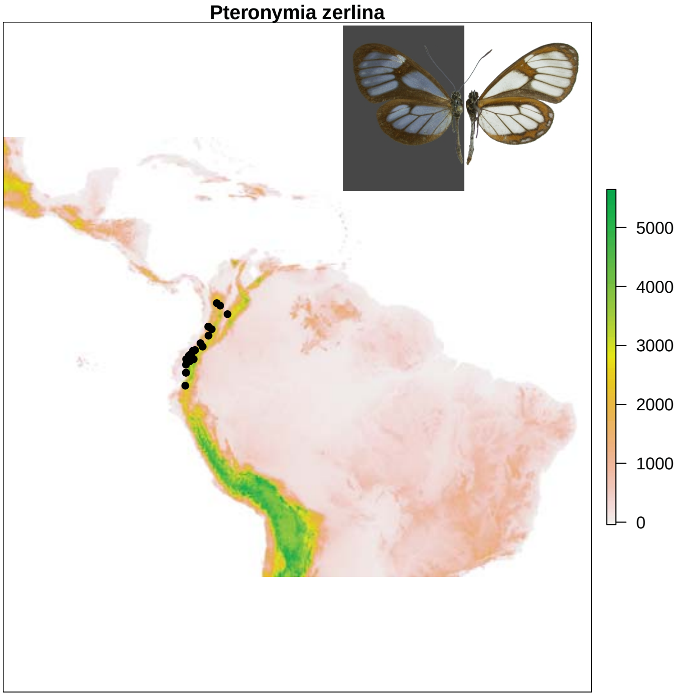

Supplementary figure S6. Summary of RASP historical biogeography inference on the 100 trees sampled from the posterior distribution (most likely states only). The figure was generated with R (https://cran.r-project.org/) and edited with Adobe Illustrator 4 (http:// www.adobe.com/uk/products/illustrator.html). The map inserted in the figure is the map from Figure 2, which was generated using ArcGIS 9.3 (http://www.edit.com/software/arcgis/).

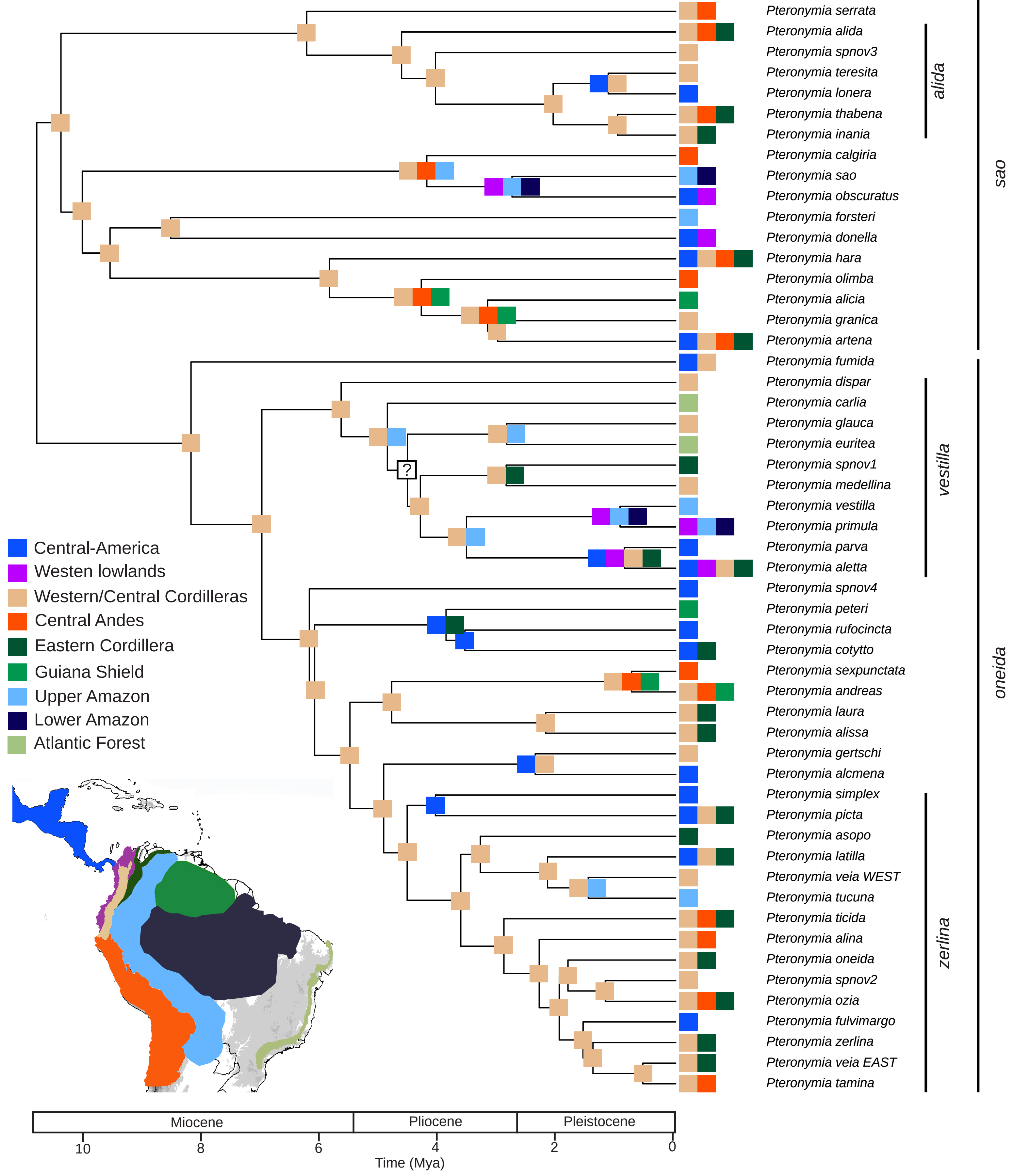

Pteronymia serrata
Pteronymia alida
Pteronymia spnov3
Pteronymia teresita
Pteronymia lonera
Pteronymia thabena
Pteronymia inania
Pteronymia calgiria
Pteronymia sao
Pteronymia obscuratus
Pteronymia forsteri
Pteronymia donella
Pteronymia hara
Pteronymia olimba
Pteronymia alicia
Pteronymia granica
Pteronymia artena
Pteronymia fumida
Pteronymia dispar
Pteronymia carlia
Pteronymia glauca
Pteronymia euritea
Pteronymia spnov1
Pteronymia medellina
Pteronymia vestilla
Pteronymia primula
Pteronymia parva
Pteronymia aletta
Pteronymia spnov4
Pteronymia peteri
Pteronymia rufocincta
Pteronymia cotytto
Pteronymia sexpunctata
Pteronymia andreas
Pteronymia laura
Pteronymia alissa
Pteronymia gertschi
Pteronymia alcmena
Pteronymia simplex
Pteronymia picta
Pteronymia asopo
Pteronymia latilla
Pteronymia veia WEST
Pteronymia tucuna
Pteronymia ticida
Pteronymia alina
Pteronymia oneida
Pteronymia spnov2
Pteronymia ozia
Pteronymia fulvimargo
Pteronymia zerlina
Pteronymia veia EAST
Pteronymia tamina
alida
sao
vestilla
oneida
zerlina
?
Central-America
Westen lowlands
Western/Central Cordilleras
Central Andes
Eastern Cordillera
Guiana Shield
Upper Amazon
Lower Amazon
Atlantic Forest
Miocene
Pliocene
Pleistocene
10
8
6
4
2
0
Time (Mya)

Supplementary figure S7. RASP historical biogeography inference on the MCC tree, all states. Sizes of colored rectangles are proportional to the support for the corresponding area as ancestral area for the node. The figure was generated with R (https://cran.r-project.org/) and edited with Adobe Illustrator 4 (http://www.adobe.com/uk/products/illustrator.html).

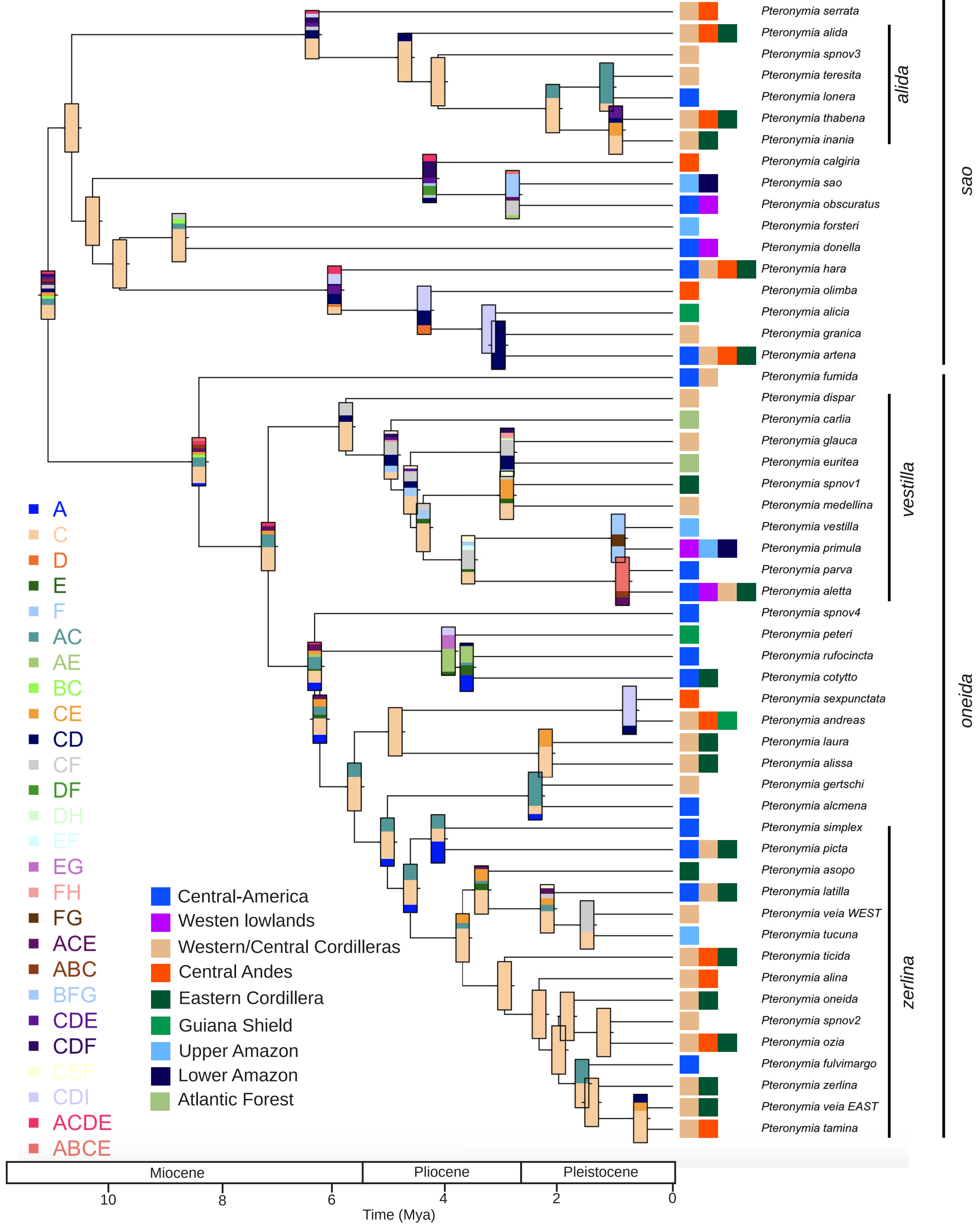

Pteronymia serrata
Pteronymia alida
Pteronymia spnov3
Pteronymia teresita
Pteronymia lonera
Pteronymia thabena
Pteronymia inania
Pteronymia calgiria
Pteronymia sao
Pteronymia obscuratus
Pteronymia forsteri
Pteronymia donella
Pteronymia hara
Pteronymia olimba
Pteronymia alicia
Pteronymia granica
Pteronymia artena
Pteronymia fumida
Pteronymia dispar
Pteronymia carlia
Pteronymia glauca
Pteronymia euritea
Pteronymia spnov1
Pteronymia medellina
Pteronymia vestilla
Pteronymia primula
Pteronymia parva
Pteronymia aletta
Pteronymia spnov4
Pteronymia peteri
Pteronymia rufocincta
Pteronymia cotytto
Pteronymia sexpunctata
Pteronymia andreas
Pteronymia laura
Pteronymia alissa
Pteronymia gertschi
Pteronymia alcmena
Pteronymia simplex
Pteronymia picta
Pteronymia asopo
Pteronymia latilla
Pteronymia veia WEST
Pteronymia tucuna
Pteronymia ticida
Pteronymia alina
Pteronymia oneida
Pteronymia spnov2
Pteronymia ozia
Pteronymia fulvimargo
Pteronymia zerlina
Pteronymia veia EAST
Pteronymia tamina
alida
sao
vestilla
oneida
zerlina
A
C
D
E
F
AC
AE
BC
CE
CD
CF
DF
DH
EF
EG
FH
FG
ACE
ABC
BFG
CDE
CDF
CEF
CDI
ACDE
ABCE
Central-America
Westen lowlands
Western/Central Cordilleras
Central Andes
Eastern Cordillera
Guiana Shield
Upper Amazon
Lower Amazon
Atlantic Forest
Miocene
Pliocene
Pleistocene
10
8
6
4
2
0
Time (Mya)

Supplementary figure S8: illustration of adult morphological characters (4 plates). Drawing were performed by Keith Willmott and Luisa Mota.

Plate 1:
A-L: Male genitalia, aedeagus, lateral view, except dorsal view in B,E,G. **M,N**: Male genitalia, lateral view. **O-V**: Male genitalia, ventral view of gnathos and inner dorsal arms of valvae.

Plate 2:
A-F: Male genitalia, lateral view. **G-K**: Male genitalia, posterior-ventral view.

Plate 3:
A-D: Male hind wing venation and androconial scales. **E-M**: Female abdomen, ventral view terminal sternites.

Plate 4:
A-G: Female genitalia, dorsal view. **H-M**: Female genitalia, ostium bursae, left lateral view, except right lateral view in K,M. **N**: Female genitalia, dorsal view, abdomen omitted.

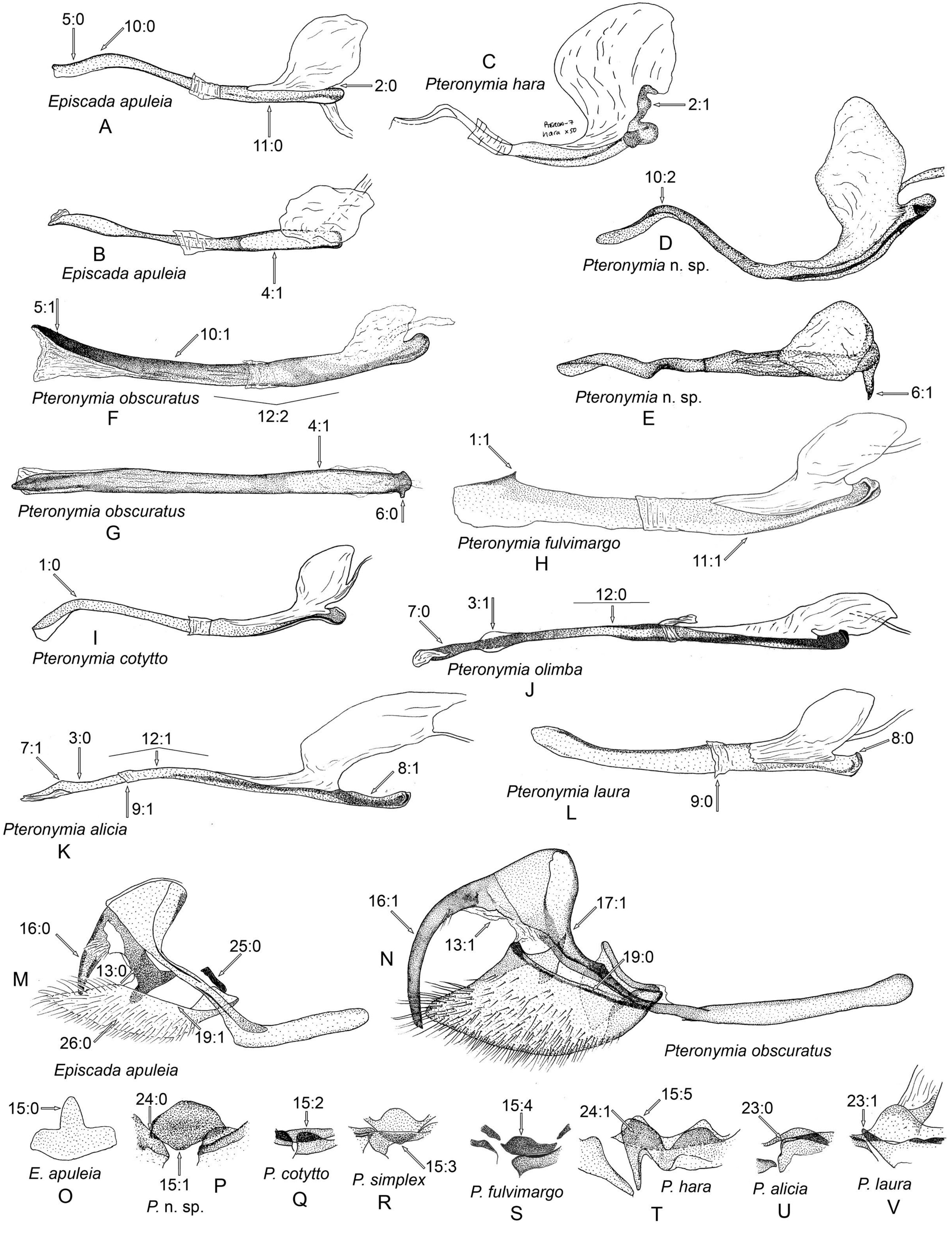
5:0
10:0
2:0
Episcada apuleia
A
11:0
C
Pteronymia hara
2:1
Episcada apuleia
B
4:1
10:2
D
Pteronymia n. sp.
5:1
10:1
Pteronymia obscuratus
F
12:2
Pteronymia n. sp.
E
6:1
4:1
Pteronymia obscuratus
G
6:0
1:1
Pteronymia fulvimargo
H
11:1
1:0
I
Pteronymia cotytto
3:1
12:0
7:0
Pteronymia olimba
J
7:1
3:0
12:1
8:1
9:1
Pteronymia alicia
K
8:0
Pteronymia laura
L
9:0
16:0
25:0
13:0
M
26:0
19:1
Episcada apuleia
16:1
17:1
13:1
N
19:0
Pteronymia obscuratus
15:0
E. apuleia
O
24:0
15:1
P. n. sp.
P
15:2
P. cotytto
Q
15:3
P. simplex
R
15:4
P. fulvimargo
S
24:1
15:5
P. hara
T
23:0
P. alicia
U
23:1
P. laura
V

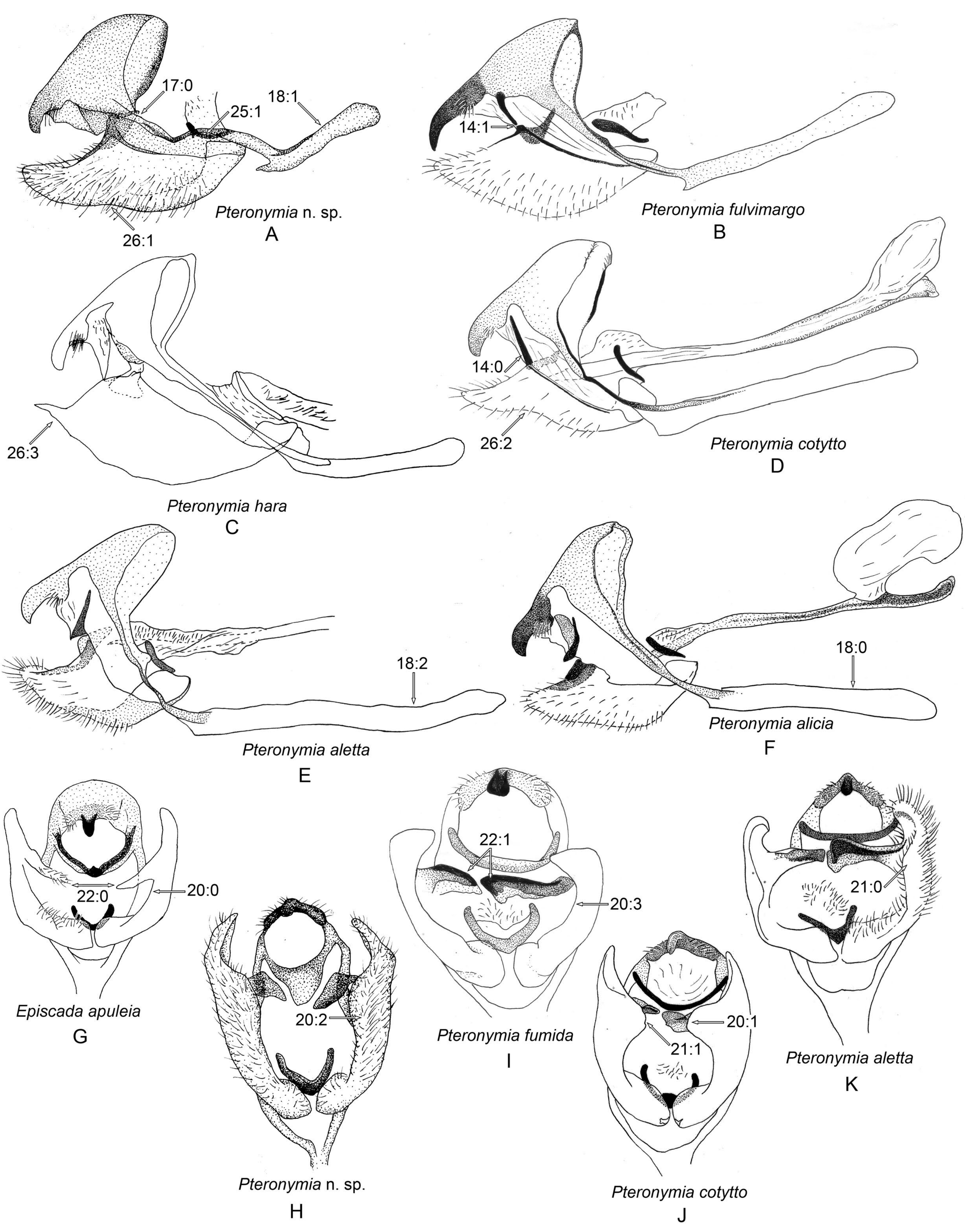
17:0
25:1
18:1
26:1
Pteronymia n. sp.
A
14:1
Pteronymia fulvimargo
B
26:3
Pteronymia hara
C
14:0
26:2
Pteronymia cotytto
D
18:2
Pteronymia aletta
E
18:0
Pteronymia alicia
F
22:0
20:0
Episcada apuleia
G
20:2
Pteronymia n. sp.
H
22:1
20:3
Pteronymia fumida
I
21:1
20:1
Pteronymia cotytto
J
21:0
Pteronymia aletta
K

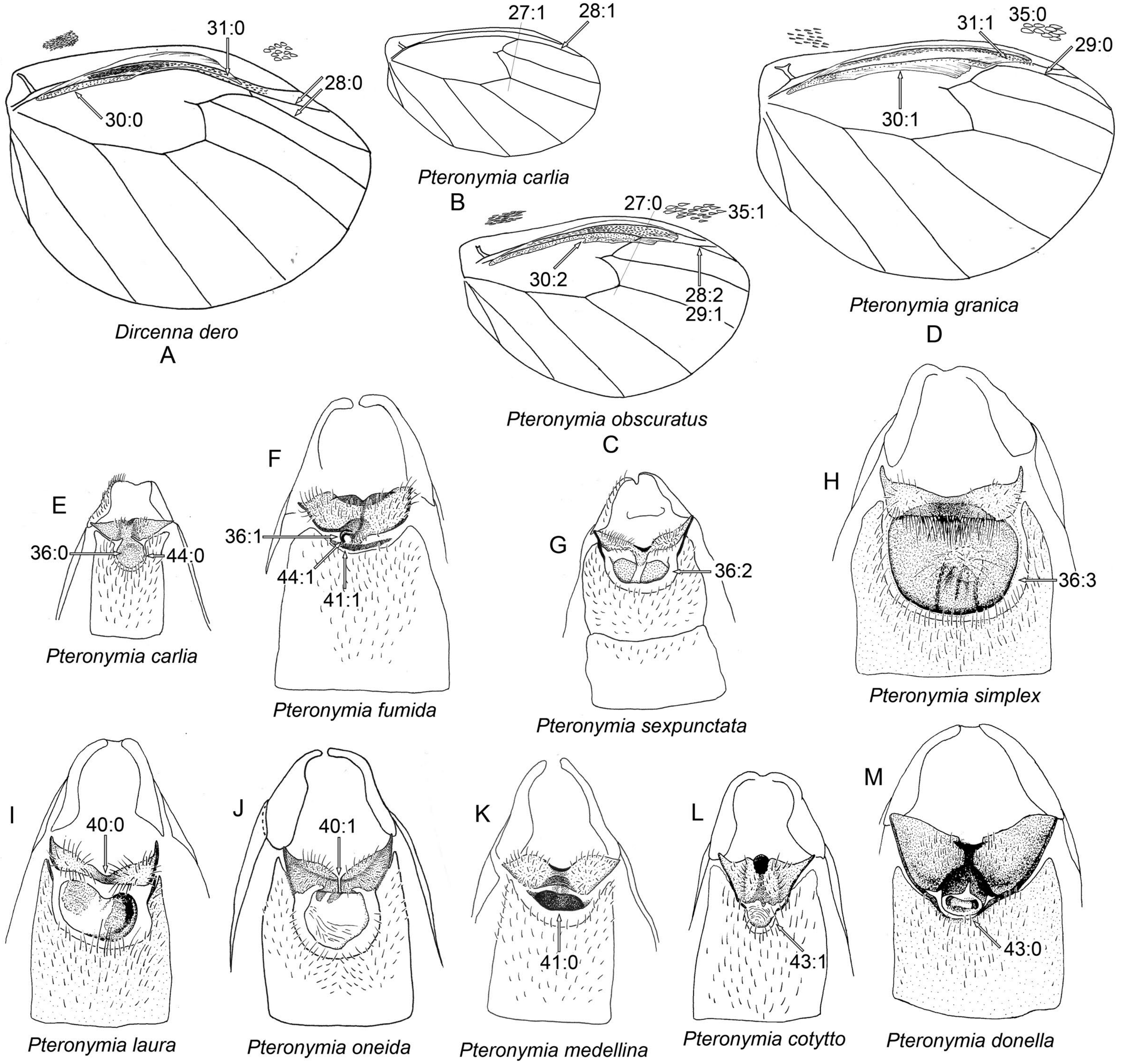
31:0
28:0
30:0
Dircenna dero
A
27:1
28:1
Pteronymia carlia
B
27:0
35:1
30:2
28:2
29:1
Pteronymia obscuratus
C
31:1
35:0
29:0
30:1
Pteronymia granica
D
E
36:0
44:0
Pteronymia carlia
F
36:1
44:1
41:1
Pteronymia fumida
G
36:2
Pteronymia sexpunctata
H
36:3
Pteronymia simplex
I
40:0
Pteronymia laura
J
40:1
Pteronymia oneida
K
41:0
Pteronymia medellina
L
43:1
Pteronymia cotytto
M
43:0
Pteronymia donella

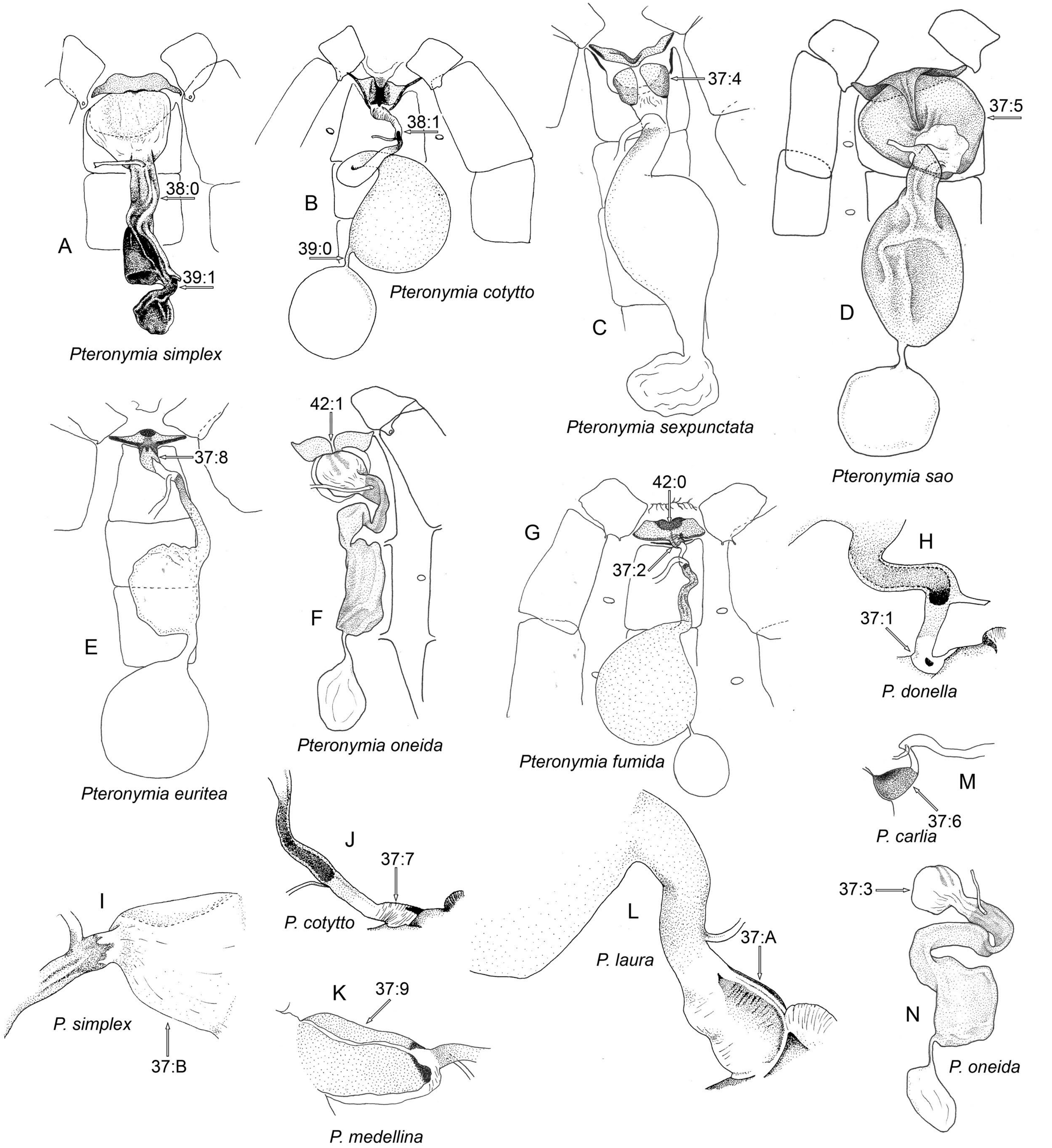
A
38:0
39:1
Pteronymia simplex
B
38:1
39:0
Pteronymia cotytto
C
37:4
Pteronymia sexpunctata
D
37:5
Pteronymia sao
E
37:8
Pteronymia euritea
F
42:1
Pteronymia oneida
G
42:0
37:2
Pteronymia fumida
H
37:1
P. donella
I
P. simplex
37:B
J
37:7
P. cotytto
K
37:9
P. medellina
L
37:A
P. laura
M
37:6
P. carlia
N
37:3
P. oneida

Supplementary figure S9: illustration of larval morphological characters
Photos were taken by André Freitas and Keith Willmott

A-I: Fifth instar larva, top dorsal view, bottom lateral view. **J-O**: Pupa, left lateral view, right dorsal view. **P,Q**: Female ventral abdomen.

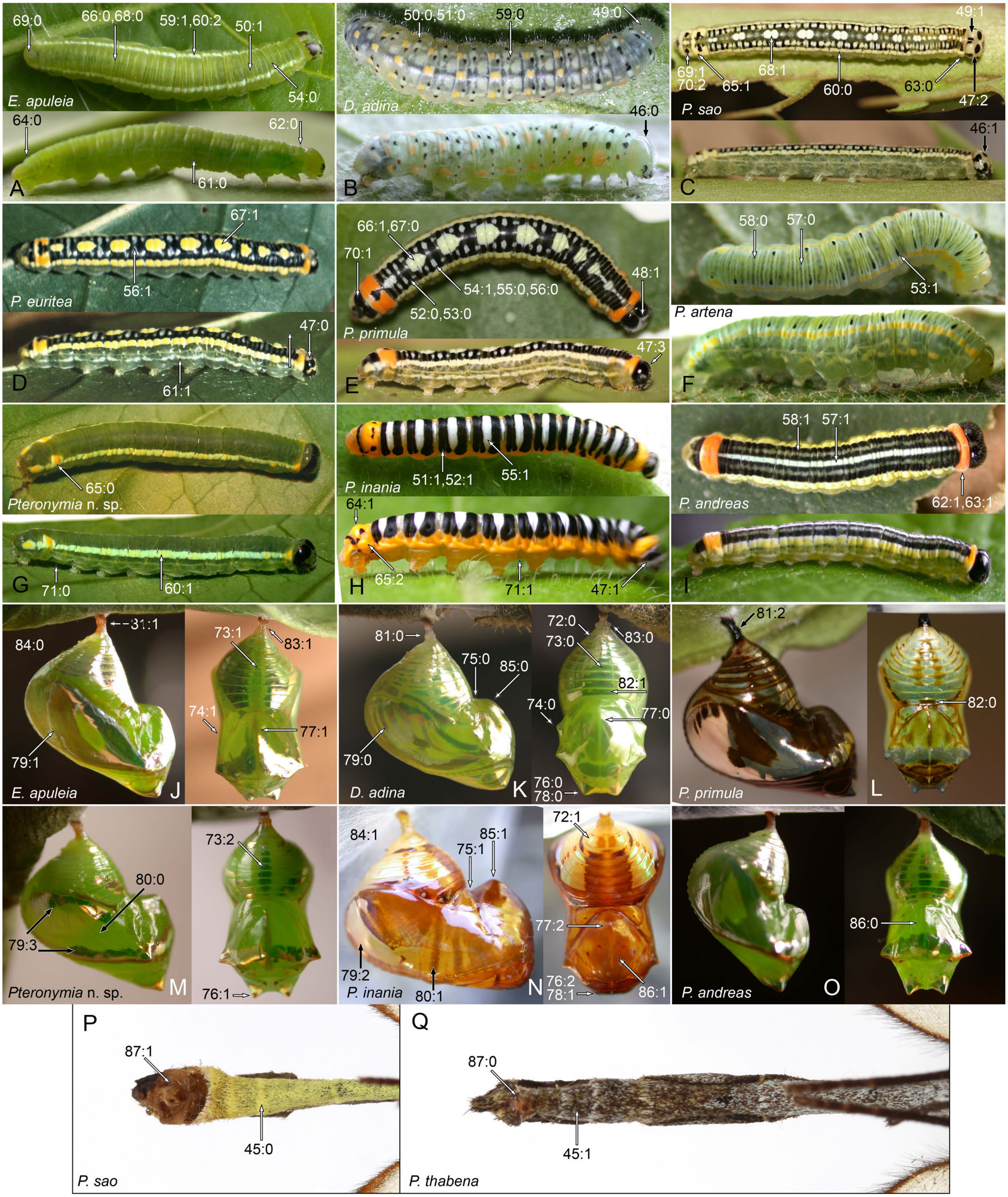
69:0
66:0,68:0
59:1,60:2
50:1
54:0
E. apuleia
64:0
62:0
61:0
A
50:0,51:0
59:0
49:0
D. adina
46:0
B
49:1
69:1
70:2
65:1
68:1
60:0
63:0
47:2
P. sao
46:1
C
67:1
56:1
P. euritea
47:0
61:1
D
66:1,67:0
70:1
48:1
54:1,55:0,56:0
52:0,53:0
P. primula
47:3
E
58:0
57:0
53:1
P. artena
F
65:0
Pteronymia n. sp.
71:0
60:1
G
P. inania
51:1,52:1
55:1
64:1
65:2
71:1
47:1
H
58:1
57:1
P. andreas
62:1,63:1
I
81:1
84:0
73:1
83:1
74:1
77:1
79:1
E. apuleia
J
81:0
75:0
85:0
79:0
D. adina
K
72:0
73:0
83:0
82:1
74:0
77:0
76:0
78:0
81:2
82:0
P. primula
L
73:2
80:0
79:3
Pteronymia n. sp.
M
76:1
84:1
75:1
85:1
79:2
P. inania
80:1
N
72:1
77:2
76:2
78:1
86:1
86:0
P. andreas
O
P
87:1
45:0
P. sao
Q
87:0
45:1
P. thabena
